# Contralateral Thermal Pain and Naturally Occurring Muscle Pain Have Different Effects on Force Production During a Fixed Perceived Effort Handgrip Task

**DOI:** 10.1002/ejp.70329

**Published:** 2026-07-08

**Authors:** Callum A. O'Malley, Thomas Mangin, Maxime Bergevin, Ilaria Monti, Christopher L. Fullerton, Alexis R. Mauger, Pierre Rainville, Benjamin Pageaux

**Affiliations:** ^1^ Department of Public Health and Sport Sciences University of Exeter Exeter UK; ^2^ Centre de Recherche de l'Institut Universitaire de Gériatrie de Montréal (CRIUGM) Montréal Canada; ^3^ École de Kinésiologie et Des Sciences de l'activité Physique (EKSAP) Faculté de Médecine, Université de Montréal Montréal Canada; ^4^ Centre Interdisciplinaire de Recherche sur le Cerveau (CIRCA) Montréal Canada; ^5^ Faculty of Health Sciences and Sport, University of Stirling Stirling UK; ^6^ School of Natural Sciences University of Kent Canterbury UK; ^7^ Département de Stomatologie Faculté de Médecine Dentaire, Université de Montréal Montréal Canada

## Abstract

**Background:**

In the presence of pain, performance during imposed workload motor tasks may be maintained at the cost of a higher perceived effort. This study investigated how experimentally induced contralateral thermal pain and naturally occurring muscle pain influence force production during a fixed perceived effort task.

**Methods:**

Forty young adults performed intermittent isometric handgrip contractions at light (13/100) or hard (50/100) perceived effort intensities across two visits. Each visit included an experimental condition, featuring contralateral thermal pain in Block 1, and a control condition, with non‐painful warm stimulation in Block 2. Participants completed 10 blocks (five repetitions of Block 1 and Block 2), with force production and electromyography continuously recorded. Participants rated thermal and muscle pain after each block.

**Results:**

Force production was higher during thermal pain compared to control, with no interaction with perceived effort intensity. Regression analysis showed that each one‐unit increase in muscle pain (0–100 scale) corresponded to a reduction in peak force (0.45 N), mean force (0.32 N) and force integral (1.02 N·s).

**Conclusions:**

During a fixed perceived effort task, contralateral thermal pain increases force production. In contrast, muscle pain is associated with reduced force production. This study suggests that the type of pain has a varied effect on force production during voluntary motor tasks. This study questions the well‐accepted inhibitory effect of pain on force production and encourages future research to use fixed perceived effort tasks to fully appraise the effects of different pain types on force production.

**Significance Statement:**

This study shows that pain‐related changes in force production during self‐regulated exercise are pain‐type dependent. Using a novel fixed perceived‐effort paradigm, we found that contralateral thermal pain increased force production, whereas naturally occurring muscle pain reduced force output. These findings extend knowledge derived from fixed‐workload tasks and suggest that distinguishing pain types may improve understanding of motor regulation in exercise, rehabilitation, and clinical contexts.

## 1 Introduction

Cognitive and physical performance is altered by pain (O'Connor and Cook 1999; Silvestrini and Corradi‐Dell'Acqua 2022). Mechanical and metabolic stimulation of A‐δ and C‐fibres elicit pain (Graven‐Nielsen 2006), thereby increasing task demand (O'Connor and Cook 1999). Pain induces neurophysiological changes that reduce the efficiency of motor command transmission to the working muscles (Rohel et al. 2021; Sanderson et al. 2021). Pain also captures attention and causes avoidance behaviours which often need to be overridden to sustain task engagement (Torta et al. 2017), increasing cognitive demand (Ecclestone and Crombez 1999). When task demands remain within one's capacity, task performance can be maintained despite pain through increased engagement of physical and cognitive resources (i.e., effort, Gendolla et al. 2019; Halperin and Vigotsky 2024; Mangin and Pageaux 2026). Consequently, during physical tasks with an imposed workload (e.g., fixed force task), performance may be maintained but with higher perceived effort (e.g., Canestri et al. 2019; Smith et al. 2020).

Our understanding of the effects of pain on task performance mostly derives from studies using experimental pain models. Amongst these, thermal pain models allow precise experimental control and localised pain stimulation. Through stimulation of thermoreceptors within the skin (Tominaga and Caterina 2004), thermal pain signals immediate tissue damage (Wilcox et al. 2018). Physical tasks can also elicit naturally occurring muscle pain, a non‐damaging, acute pain experienced only during physical exercise (Cook et al. 1997). Both pain types have been suggested to increase cognitive and physical task demands (O'Connor and Cook 1999; Silvestrini and Corradi‐Dell'Acqua 2022; Wilcox et al. 2018), potentially causing participants to disengage earlier or report higher perceived effort to maintain task performance (O'Connor and Cook 1999; Silvestrini and Corradi‐Dell'Acqua 2022).

Physical task performance, also referred to as human physical performance is the measurable output of a human action, quantified using metrics such as time, power, or force. (Briand et al. 2025). Foundational knowledge on the effects of pain on physical task performance primarily derives from studies using fixed‐workload tasks performed until task disengagement. In these tasks, force or power output is fixed, requiring participants to maintain a constant workload. Such studies suggest that pain decreases task performance, as participants disengage earlier than under non‐pain conditions (Aboodarda et al. 2020; Canestri et al. 2019; Norbury et al. 2022a). Other foundational studies using fixed‐workload tasks not performed until task failure identified neurophysiological and biomechanical adaptations supporting force maintenance. For example, experimental pain increases force variability (Arvanitidis et al. 2025), alters load sharing amongst synergistic muscles (Hug et al. 2014), and redistributes electromyographic activity (Falla et al. 2017). While fixed‐force tasks provided valuable insight into how pain affects force production in controlled settings, where force output is imposed and neurophysiological and biomechanical adaptations support maintenance of the target force, they do not allow investigation of how humans adapt to pain when free to regulate their own force production.

Another paradigm emerging from the sports science literature to evaluate task performance is the use of fixed perceived effort tasks. In these tasks, individuals are free to regulate force production to maintain a constant perceived effort intensity (O'Malley et al. 2023). As human behaviour is thought to depend on effort and its perception (e.g., Inzlicht et al. 2018; Preston and Wegner 2009), including within the physical domain where effort perception influences both physical task performance (e.g., Marcora and Staiano 2010; Pageaux 2014) and the regulation of force, movement and physical activity (e.g., Mangin and Pageaux 2026; Cheval and Boisgontier 2021), fixed perceived effort tasks are particularly relevant for understanding how pain interacts with the self‐regulation of force production. These tasks provide a useful framework for reflecting how humans regulate everyday movement responses to pain (Arendt‐Nielsen and Graven‐Nielsen 2008; Preston and Wegner 2009). Consequently, fixed perceived effort tasks offer a valuable method to investigate whether pain type differentially influences the self‐regulation of force.

In this context, this preregistered study aimed to investigate the effect of experimentally induced thermal pain and naturally occurring muscle pain on force production during an isometric handgrip task performed at a fixed perceived effort. We hypothesised that, during the fixed perceived effort task, participants would produce less force in the presence of contralateral thermal pain than in the presence of a non‐painful thermal stimulation. We also hypothesised that force production would decrease in the presence of naturally occurring muscle pain. As exercise intensity was manipulated and given that naturally occurring muscle pain increases with exercise intensity, we further hypothesised that higher pain ratings during high‐intensity exercise would result in a greater decrease in force production compared with low‐intensity exercise.

## 2 Methods

### 2.1 Participants

Forty healthy active participants volunteered to this study (mean ± SD, age: 25.1 ± 3.8 years; 50% each sex). Further participant details can be found in Table S1. A sensitivity analysis performed with G*Power software (version 3.1, G*Power: Germany) showed that a sample size of 40 participants could detect moderate‐large effect sizes of $f\left(U\right)$ = 0.310 (equivalent to $\eta_{p}^{2}$ = 0.088) with a statistical power of β = 0.80, ⍺ = 0.05, for within‐participants comparisons with four measurements, and with the ‘as SPSS’ option selected. Participants had no contraindications to physical exercise and were free from existing pain and known chronic conditions. Before each visit, participants were asked (i) to abstain from food (2 h), caffeine (4 h), vigorous physical exercise (24 h), alcohol (24 h), cannabis (48 h), analgesics (48 h), and (ii) to repeat their eating and drinking habits prior to each laboratory visits. Participants were compensated $75 CAD at the completion of the third visit for their time.

### 2.2 Overview of the Study

The study was preregistered on Open Science Framework (https://osf.io/8tbm3/). Ethical approval was obtained from the local ethics committee (CER VN‐22‐23‐16). Participants provided written informed consent prior to any data collection and all procedures were conducted in accordance with the Declaration of Helsinki. All recruited participants completed the study and all data were included for analysis. The ambient conditions of the laboratory were controlled at ~18.0°C and ~60% humidity for all participants and visits.

**FIGURE 1:**
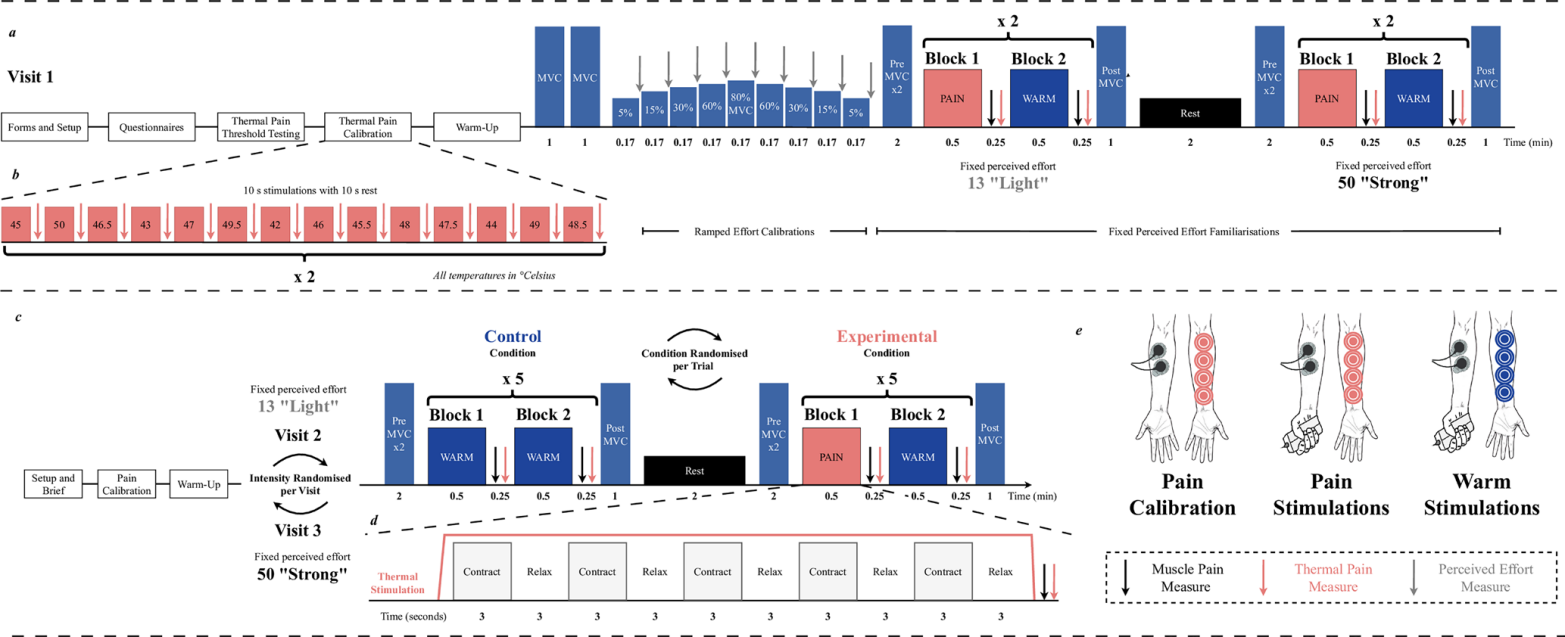
Visual representation of research procedures with inbuilt legend and timings. Panel a shows a broad overview of Visit 1 familiarisation and calibration. Panel b depicts the thermal pain calibration procedure. Panel c shows a broad overview of Visits 2 and 3 involving the experimental trials which were randomised for fixed perceived intensity (light vs. strong) between visits as well as experimental conditions between trials within visits. Panel d depicts the intermittent isometric handgrip procedure during the experimental Block 1 with a painful thermal stimulation rising and returning to a standard warm (40°C) control temperature at all other times. Panel e visualises the stimulation sites on the anterior surface of the non‐dominant arm, electromyography electrode placement on the anterior surface of the dominant arm, and handgrip placed in the dominant hand. Colours of red (pain) and blue (warm) are corresponded to the main depictions in Panel a and c. Muscle pain, thermal pain, and perceived effort ratings are represented by the inbuilt legend.

The study employed a repeated measure, split‐plot, crossover design. Participants visited the laboratory on three occasions. The laboratory visits were spaced 2–7 days apart. In visit 1, participants completed a thermal pain calibration (see Section 2.4) and familiarisation with the handgrip dynamometer (see Section 2.3), the fixed perceived effort task and all other procedures. In visits 2 and 3, participants were asked to perform a fixed perceived effort task consisting of 50 isometric contractions at a light or strong effort (see Section 2.3.1). To do so, the participants were asked to experience a light or strong perceived effort intensity during each handgrip contraction. Fixed perceived effort intensities were pseudorandomised and counterbalanced between visits. Within visits 2 and 3, participants completed an experimental and control condition in a pseudo‐randomised and counterbalanced order. The control condition only involved warm (non‐painful) control stimulations during the fixed perceived effort task, whereas the experimental condition involved an alternation between painful thermal stimulations and warm control stimulations during the same task. Participants completed handgrip maximal voluntary contractions (MVC) before and immediately after completion of the fixed perceived effort task. A detailed overview of the study is presented in Figure 1.

#### 2.2.1 Visit 1—Calibration and Familiarisation Trials

Upon arrival to the laboratory, a researcher described the main aim of the study was to investigate the effect of pain on motor behaviour. The protocols of the study were explained, and participants asked any questions before completing a pre‐study screening questionnaire, a consent form, the Dijon physical activity score, and the Edinburgh handedness questionnaire (Materials S1 and S2). Following this, participants were asked to complete a 15‐item task‐specific motivation questionnaire, a 13‐item pain catastrophising scale, and a 40‐item five facets of mindfulness questionnaire (Materials S3–S5). The researcher then instructed the participant to fit a heart rate monitor and a respiratory belt to their torso. Lastly, the researcher prepped the dominant arm for the attachment of electrodes for sampling electromyographic traces as well as marking four equally spaced sites on the non‐dominant arm for the thermal probe to be placed.

After the pain threshold and calibration testing (see Section 2.4.1 and 2.4.2), participants performed a short warm‐up comprising 10 contractions at what the participant considered to be 50% of their maximum force. Contractions and rest periods lasted approximately 3 s each and were prompted by verbal ‘GO – STOP’ instructions for the start and end of each handgrip contraction. The instructions were provided by pre‐recorded audio prompts (see Section 2.3.1). Immediately after the warm‐up, participants completed two MVCs. Participants then completed a pyramidal effort calibration task. Participants were asked to squeeze the handgrip for 10 s at an intensity which corresponded to 5%–15%–30%–60%–80%–60%–30%–15%–5% of their MVC peak force. Participants were shown real‐time force data from the handgrip. Participants were instructed that the force trace must remain within a green band (±2% of the target force percentage) for the entire 10‐s period for the trial to be considered valid and complete. After each 10‐s contraction, participants were asked to relax and report the intensity of the effort perceived at the end of the contraction (see Section 2.5.1). This was followed by a 30‐s rest and the next intensity of contraction. The pyramidal effort calibration task was completed to allow the participant to squeeze the dynamometer at different force levels, thus experiencing various intensities of effort during handgrip contractions.

Participants were then familiarised with the intermittent isometric handgrip task at a fixed light and strong perceived effort. Before the fixed perceived effort task, participants completed two MVCs. The intensity of perceived effort light and strong was provided via the verbal anchors of the category‐ratio (CR) 100 scale (see Section 2.5.1). The familiarisations with each intensity lasted approximately 5 min each, including completion of the MVCs and effort ratings. Participants completed first the intensity light and then the intensity strong. This familiarisation involved a shortened version of the main experimental task with only two repetitions of each block (20 contractions) instead of five, including the warm and painful stimulations as completed in the experimental condition (see Section 2.5.2).

After the completion of every five contractions (i.e., one block), the participants rated there: (i) naturally occurring muscle pain in their dominant forearm; and (ii) thermal pain or warm sensation in their non‐dominant arm. Participants also performed two handgrip MVCs before the perceived effort task, and one MVC immediately after.

#### 2.2.2 Visit 2 and 3—Experimental Visits

The visits 2 and 3 involved the same setup and preparation of equipment and instructions as visit 1. Following the pain calibration, participants completed an identical warm‐up to visit one. After this, participants were informed about the prescribed perceived effort intensity (light, 13; or strong, 50) for the intermittent handgrip task within the select visit. Participants were not informed which condition would be completed first. Prior to completing the first condition, participants completed the motivation, boredom and fatigue visual analogue scales (VAS, see Section 2.5.3) and performed MVCs before starting the fixed perceived effort task. Immediately after completion of the perceived effort task, participants performed another MVC, followed by 2 min passive rest. At the end of the rest period, participants then completed the motivation, boredom, and fatigue VAS. Then, participants were informed via the automated instruction set that they were about to begin the next condition at the same fixed perceived effort intensity. Participants completed the next condition in an identical fashion to the first condition, including the post MVC. Lastly, participants completed a final motivation, boredom and fatigue VAS.

In the next experimental visit (visit three), participants completed exactly the same protocols except at a different fixed perceived effort (e.g., if visit 2 = light effort, visit 3 = strong effort, and *vice versa*). At the end of the last visit, participants were debriefed about the full aims of the study, provided their details for monetary compensation, and then exited the laboratory.

### 2.3 Handgrip Tasks

Throughout all visits, participants sat in an adjustable chair facing a monitor positioned approximately 70 cm in front of them at eye‐level and both arms resting on the table. Participants sat upright with their back resting on the chair support and forearms resting on the table in front of them with elbows at approximately 90°–100° flexion. Forearms were in a supinated position and the handgrip (MLT004/ST, ADInstruments, New Zealand) was placed in the dominant hand. For all handgrip contractions, participants were asked to squeeze the handgrip without changing the angle of their wrist, elbow, shoulder, or hips (i.e., leaning into the contraction). No feedback of the force was provided to the participants except during the familiarisation in visit 1.

The handgrip dynamometer was connected to a PowerLab 16/35 system (ADInstruments, New Zealand). The force signal was digitised online at a sampling frequency of 2 kHz using a computer and stored for analysis with commercially available software (LabChart8 Pro software, ADInstruments, New Zealand).

#### 2.3.1 Fixed Perceived Effort Task

The fixed perceived effort task consisted of an intermittent handgrip contraction task, consisting of 3 s contraction and 3 s rest. An intermittent structure for the task was chosen to avoid the mechanical compression occurring during a sustained isometric contraction, which inevitably limits the local blood flow even for contractions as low as 20% of MVC peak torque (Lind 2011). Such mechanical compression would create excessive ischaemia that would exacerbate naturally occurring muscle pain due to accumulation of metabolites in the muscle (Aboodarda et al. 2020).

Participants performed a fixed perceived effort task prescribed with the CR100 scale (Borg and Borg 2002) according to a fixed light (13) or strong (50) perceived effort intensity. Each condition involved two blocks (Block 1 and Block 2). During the experimental condition, Block 1 involved a painful contralateral thermal stimulation eliciting a ‘strong’ thermal pain rating at rest (see Section 2.4.3). During the control condition, Block 1 involved a control stimulation at a warm temperature of 40°C—below the 42°C threshold of skin thermoreceptors (Tominaga and Caterina 2004). For Block 2, the thermal stimulation was always a warm control stimulation of 40°C in both conditions. Blocks 1 and 2 were repeated five times, meaning that a total of 10 blocks (five of Block 1 and five of Block 2) were performed within each condition. Within each block, participants performed five isometric handgrip contractions.

The start and end of each contraction was signalled to the participants via pre‐programmed audio instructions using SuperLab6 software (Cedrus, USA). A countdown of 3–2–1 was shown on the monitor in front of the participants before the first audio prompt to contract (‘GO’) followed 3 s later by a prompt to relax (‘STOP’). All audio prompts were separated by 3 s until five contractions were performed. After the last ‘STOP’ prompt, there was an 18‐s period in which participants rested and provided verbal pain intensity ratings. Participants were instructed to contract and relax in relation to the audio prompts, by reacting as quickly as possible. After the initial countdown, and therefore, during each contraction, participants only had the CR100 scale displayed on the monitor. No feedback of the force or other physiological measures (see Section 2.6) was provided.

#### 2.3.2 Maximal Voluntary Contractions

Before and after each fixed perceived effort task, participants completed handgrip MVCs to measure their maximal force production capacity. Before the fixed perceived effort task, if the peak force of the second MVC was > ±10% from the first MVC, a third MVC was performed in order to secure the obtention of the true maximal voluntary force. Each MVC was separated by at least 1 min of passive rest. After completion of the perceived effort task, participants performed a single MVC. Participants were instructed ‘to squeeze the handgrip as hard as you can for 3 s’. Verbal encouragement was provided during all MVCs.

### 2.4 Thermal Pain Stimulation

Thermal stimulations were applied via a thermosensory stimulator and a nine cm^2^ rectangular probe (QST Lab, Strasbourg, France). All stimulation zones on the probe were programmed to emit the same temperature. A researcher positioned the probe on one of four equally spaced sites on the anterior surface of the participants' non‐dominant (contralateral) forearm. Sites were marked with numbers: 1—closest to the wrist, to 4—closest to the olecranon fossa. To limit temporal summation of pain at each site, researchers pre‐randomised and counterbalanced the order of stimulation sites for the familiarisation and experimental trials, which avoided successive stimulations to any site and an equal number of stimulations to each site across the visit.

#### 2.4.1 Thermal Pain Threshold

During a thermal pain threshold assessment (Figure 1a) at each of the four forearm sites, the thermal probe was placed on the skin emitting a temperature of 37.5°C which all participants confirmed was warm, but not painful. When participants indicated they were ready, the thermal probe increased in temperature at 0.2°C.s^−1^. Throughout, participants held a button in their dominant hand and were asked to click the button when they considered that the thermal stimulation changed from being a warm stimulation to a ‘hot, painful’ stimulation—that is, first change in a rating from 0 on the CR100 scale (see Section 2.5.2). Whereupon the participant clicked the button, the thermal stimulation dropped immediately back to 37.5°C at a rate of 300°C.s^−1^ and the researcher removed the probe from the skin. The same procedure was repeated at each stimulation site with 1 min rest between each test. Researchers noted the temperature for each assessment as a measure of thermal pain threshold.

#### 2.4.2 Thermal Pain Calibrations

Participants then underwent a thermal pain calibration test. Participants received a randomised sequence of 14 thermal stimulation intensities for 10 s each. Thermal stimulations ranged from 42°C to 45°C as well as every 0.5°C between 45°C and 50°C (Figure 1b). After each 10 s stimulation, the thermal probe was lifted off the skin and participants had a 10 s rest period to verbally report their intensity of thermal pain to the researcher. If participants considered the thermal stimulation as above their tolerance, they were asked to indicate this to the researcher who lifted the probe off the arm. In this case, the lead researcher denoted the thermal pain rating as 100 ‘maximal’. The thermal probe was reapplied to the skin 2 s before the next stimulation. Immediately after 10 s rest, the next stimulation was applied with a 300°C.s^−1^ rate change in stimulation intensity. A pre‐randomised order determined which forearm site the thermal probe was applied to with each site stimulated seven times. Each thermal pain rating was recorded and plotted using Microsoft Excel. A logarithmic regression determined which temperature corresponded to a rating of 50 ‘strong’ for thermal pain on the CR100 scale. This temperature was rounded to the nearest 0.5°C and used as the temperature for the familiarisation and the first temperature for the pain calibration to take place at the onset of each subsequent visit.

During experimental visits, participants completed a short recalibration to the thermal pain stimulations (Figure 1c). This calibration involved a 30 s stimulation of one of the four sites on the non‐dominant forearm before the fixed perceived effort task. This 30 s duration was chosen as it corresponds to the duration of the thermal stimulation applied concomitantly to the 5 intermittent handgrip contractions of the perceived effort task. The first temperature that was used was the regressed temperature from the pain calibration in the familiarisation visit. Participants were asked to rate their thermal pain at the end of the 30 s. If the rating was above/below 50 ‘strong’, then the research adjusted the next stimulation by 0.5°C and completed another 30 s stimulation at a different stimulation site until a rating of 50 ‘strong’ was given. If participants did not provide a rating of 50 ‘strong’ after five stimulations, the temperature with the closest rating to 50 was used as the stimulation intensity for the upcoming experimental task.

#### 2.4.3 Thermal Pain During Fixed Perceived Effort Task

During each experimental visit, an equal number of five stimulations were applied to each site. The thermal probe was placed on the skin site 3 s before each block and removed 3 s after the last contraction of each block (Figure 1d). Thermal stimulation intensity was set at 40°C at all times (control condition and experimental condition Block 2), except during the experimental condition Block 1 when thermal stimulation intensity was programmed according to a prior pain calibration (mean ± SD = 46.7°C ± 1.1°C, range = 44°C–50°C). Onset of thermal stimulations during experimental condition Block 1 lasted 32 s with an onset 1 s before the first contraction and offset 1 s after the last relaxation. Rate change in thermal stimulation intensity was set to 300°C.s^−1^.

### 2.5 Psychological Variables

#### 2.5.1 Perceived Effort

The intensity of perceived effort was reported in visit 1 during the pyramidal effort calibration task (see Figure 1a) and used for exercise prescription in all visits, using the CR100 scale (Borg and Borg 2002, see Material S6). The perception of effort was defined as ‘how hard, heavy and strenuous the physical task is’ (Marcora 2009). During the pyramidal effort calibration task (see Section 2.2.1) participants rated their perceived effort at the end of the last contraction. During the fixed perceived effort tasks, participants were instructed to consider the intensity of their perceived effort on a moment‐by‐moment basis and adjust the intensity of their handgrip contraction to maintain a fixed perceived effort. The lower and uppermost anchors of the CR100 scale were described according to the handgrip task (Halperin and Emanuel 2020; Pageaux 2016). The lowermost boundary of 0 ‘nothing at all’ related to when the participant was rested with the handgrip resting on the palm of the hand but not squeezing, and the context‐related uppermost boundary of 100 ‘maximal’ was related to when the participants completed an MVC where they squeezed as hard as they could (i.e., maximal effort).

#### 2.5.2 Naturally Occurring Muscle Pain and Contralateral Thermal Pain

Naturally occurring muscle pain ratings in the dominant arm and thermal pain ratings in the non‐dominant arm were recorded using the CR100 scale. Pain was defined to the participants as ‘the degree of hurt you perceive’ (Hawker et al. 2011). Naturally occurring muscle pain focused on the degree of hurt perceived in the working musculature (dominant forearm). Thermal pain related to the degree of hurt perceived from thermal stimulation of the skin (non‐dominant forearm).

Lower and uppermost boundaries were predefined for each pain rating. For instance, the lowermost boundary of 0 ‘nothing at all’ was related to how participants felt during rest without any exercise or thermal stimulation. The uppermost boundary of 100 ‘maximal’ denoted the maximal tolerable pain level that a participant could deal with at that moment. In relation to naturally occurring muscle pain, this would refer to the point where the exercise task elicited an intensity of pain that would exceed the pain tolerance and cause the individual to withdraw from the exercise. In relation to thermal pain, this would refer to the point at which the individual would require the thermal probe to be removed from the skin. Participants were always instructed to consider their pain ratings from the end of the latest contraction/stimulation. Participants were also explicitly instructed to report a rating of 0 if the thermal stimulation generated warm and non‐painful sensations.

Perceived effort and pain could be rated on the same scale, as far as instructions given to the participant explicitly dissociate both constructs (Halperin and Emanuel 2020; Pageaux 2016; Pageaux et al. 2020). For instance, an individual could be rested (i.e., not squeezing the handgrip) and therefore not perceive any effort applied to the task but simultaneously receive a thermal stimulation causing pain. On the other hand, an individual could complete a very low intensity isometric handgrip for a short duration which would cause them to perceive some effort applied to the task but not involve a thermal stimulation meaning they would not perceive any thermal pain and unlikely any naturally occurring muscle pain due to the low intensity of the exercise (Cook et al. 1997).

#### 2.5.3 Motivation, Boredom and Fatigue

Motivation, boredom and fatigue are known to interact with effort and performance (Mangin and Pageaux 2024). In each visit, before the first condition (pre), before the second condition (mid), and after the second condition (post), participants rated their motivation, boredom and fatigue using visual analogue scales (VAS). The details of each VAS are available in Materials S7–S9. Participants were asked to draw a vertical marker on three 100 mm VAS. Responses in mm were recorded from the left part of the scale to the mark indicated by the participant.

### 2.6 Physiological Variables

#### 2.6.1 Electromyography

During all familiarisation and experimental trials, the electromyographic (EMG) activity of the flexor carpi radialis (agonist) and extensor carpi radialis (antagonist) muscles was recorded through two pairs of 10 mm diameter, silver‐chloride circular surface electrodes (Cardinal Health, USA). Following SENIAM guidelines, the sites were shaved, exfoliated, and cleaned with alcohol swabs before placing the electrodes onto the skin with a 20 mm space between each electrode (Hermens et al. 2000). A reference electrode was placed on the olecranon process of the dominant arm. Commercially available shielded lead wires (ADInstruments, New Zealand) from the surface electrodes were taped to the table away from other sources of interference. Myoelectrical signals were digitised through LabChart8 Pro software at a sampling frequency of 2 kHz. Traces were amplified with a bandwidth frequency ranging from 1 Hz to 5 kHz, and a notch filter centred at 60 Hz was applied. Myographic traces were sampled in millivolts (mV) with a root mean square (RMS) automatically calculated during data extraction from the LabChart8 Pro software. A normalised value was also calculated as a percentage of the RMS from pre‐trial MVC with the highest peak value. Co‐contraction index measures were also calculated (Equation 1, Knarr et al. 2012) according to raw and normalised EMG activity.

(1) $$\;\mathrm{co}-\text{contraction index}=\text{antagonist}/\left(\text{agonist}\;+\;\text{antagonist}\right)$$

#### 2.6.2 Heart Rate

Heart rate data were obtained via a heart rate monitor (T31, Polar, Finland) worn on the chest and continuously sampled through LabChart8 Pro software. Digital heart rate signals were sampled into one channel of the LabChart8 Pro software, and another channel ran automatic cyclic calculations to determine the heart rate frequency (beats.min^−1^).

#### 2.6.3 Respiration

Respiratory frequency data were obtained via a respiratory belt (TN11132/ST, ADInstruments, New Zealand) worn over the diaphragm and sampled through LabChart8 Pro software using a Bluetooth receiver. Digital respiratory frequency signals were continuously sampled into one channel of the LabChart8 Pro software, and another channel ran automatic cyclic calculations to determine respiratory frequency (breaths.min^−1^).

### 2.7 Data Analysis

Data analysis was completed with the LabChart 8 Pro software. Force traces were filtered using a low‐pass Butterworth filter of 5 Hz prior to data analysis (Maffliuetti et al. 2016). The peak force, mean force, force‐time integral, and peak rate of force development (RFD) were extracted for all MVC and intermittent contractions. The peak force and peak RFD values were normalised by the highest peak value obtained during the MVCs completed before each fixed perceived effort task, then expressed as a percentage. Mean force and force‐time integral data were not normalised due to varying contraction durations.

Contraction onset and offset were automatically detected with the Peak Analysis module of LabChart8 Pro, using a 2SD change in raw force threshold, from a 2 s resting baseline before/after each contraction. Reaction times to contract and relax audio prompts and contraction duration were calculated for each fixed perceived effort contraction. If any reaction time was ≤ 140 ms (Ollman and Billington 1972), the response was marked as a ‘false start’, and the reaction time was removed from the spreadsheet. The number of false starts was calculated for both contract and relax prompts. The reaction time to the first contract prompt was removed from analysis as the participants received anticipatory cues on screen for when the first contraction would begin. Therefore, only contractions two to five of each block were included in the analysis of false starts and reaction time to the contraction prompt. Root mean square (RMS) of the flexor carpi radialis and extensor carpi radialis EMG signal, mean heart rate, mean breathing frequency, and the temperature of the thermal stimulation during each contraction were calculated by the LabChart8 Pro software.

All participants' data were averaged according to the: intensity (light vs. strong); condition (control vs. experimental); block (Block 1 vs. Block 2); and repetition (repetition 1 vs. 2 vs. 3 vs. 4 vs. 5), thus creating 40 columns of data relating to each of these factors (e.g., raw peak force for light, control, Block 1, repetition 1).

### 2.8 Statistical Analysis

#### 2.8.1 A Priori Analysis

Averaged and compiled data were then exported to JASP (version 0.19) and JAMOVI (version 2.3.28) software for subsequent statistical analysis. Repeated measures ANOVAs were performed with the following factors: perceived effort intensity (light vs. strong); condition (control vs. experimental); block (Block 1 vs. Block 2); repetition (repetition 1 vs. 2 vs. 3 vs. 4 vs. 5). Greenhouse–Geisser corrections were applied to data that violated sphericity.

Consistent with our pre‐registered hypotheses, and to isolate the specific effect of thermal pain (only Block 1 of the experimental condition) on force production and electromyography signal, we focused primarily on the condition × block interaction. Additionally, an intensity × condition × block interaction was investigated to identify whether any changes in force parameters due to the experimental or control conditions varied between light and strong fixed perceived effort intensities.

All main and interaction effects were assessed at an alpha level of p ≤ 0.05 (two tailed). Main effects were reported with F values, partial eta squared ($\eta_{p}^{2}$) and upper and lower 95% confidence interval (CI) boundaries. Post hoc pairwise comparisons were completed in JASP and JAMOVI and reported with t values, Cohen's d, and upper and lower 95% CIs. Multiple comparisons were adjusted with the Holm‐Bonferroni correction.

#### 2.8.2 Exploratory Analysis

In addition to repeated measures ANOVAs, two linear regression analyses were completed to test the effect of naturally occurring muscle pain on force production. This was completed with two linear regression analyses. The first linear regression analysis involved muscle pain ratings and intensity as two factors to judge their effects on force production. As we recruited an equal number of each sex, and because there is evidence of sex on pain perception (Cook et al. 1998), we included this as an additional factor in the regression model as sex may influence pain perception. The inclusion of sex as a factor is further justified by a sex difference in muscle pain ratings assessed via a Mann–Whitney U test.

We also controlled for the induction of fatigue by measuring changes in peak force from the MVCs pre‐ to post‐condition. Repeated measures ANOVAs were completed for the MVC values to assess neuromuscular fatigue‐related effects from the experimental bouts. The MVC data were categorised according to intensity, condition and time‐point (e.g., pre‐fixed perceived effort task vs. post‐ pre‐fixed perceived effort). Similarly, VAS ratings for motivation, boredom, and fatigue were entered into a 2 (intensity) × 3 (pre‐, mid‐, post‐trial) repeated measures ANOVA.

As fatigue was identified as an important confounding factor, a second exploratory linear regression analysis was conducted using a three‐step model where muscle pain ratings were entered as the primary factor, followed by exercise intensity, and pre‐to‐post changes in MVC peak force (objective fatigue) and fatigue VAS ratings (subjective fatigue) as covariates. This approach accounted for the effects of fatigue on force production. Unlike the first regression model, which averaged force production across each block, the second model averaged force production across all conditions, as fatigue measures were only collected before and after each condition.

Any further exploratory analysis is available in the Supporting Information. In which, condition × block interactions of heart rate, breathing frequency, and behavioural parameters (e.g., false starts, reaction times, and contraction duration) were conducted. Comparisons over time‐on‐task (i.e., pairwise comparison of repetitions) were considered. Specifically, we explored the difference between the first and all subsequent repetitions (e.g., 1 vs. 2, 1 vs. 3, 1 vs. 4, 1 vs. 5) as well as the difference between the last and all previous repetitions (2 vs. 5, 3 vs. 5, 4 vs. 5). An additional condition × block × repetition effect explored whether time‐on‐task influenced the contralateral thermal pain‐related effects on dependent variables. Finally, the intensity × condition × block × repetition interaction was examined to assess whether task intensity influenced the contralateral thermal pain‐related intervention effect over time‐on‐task.

Lastly, averaged contralateral thermal pain ratings from the resting calibration period and each repetition (i.e., 1 through to 5) within the experimental Block 1 only were entered into a repeated measures ANOVA to assess any potential exercise‐induced hypoalgesia effects to the thermal stimulation. All repetitions for thermal pain ratings in experimental Block 1 were also averaged per intensity and a student t test was completed between baseline and experimental thermal pain ratings for each intensity. An additional t test was completed between each intensity's mean thermal pain ratings in Block 1 to determine if perceived exercise intensity had an impact on exercise‐induced hypoalgesia.

## 3 Results

Details of all ANOVAs results performed are available in Table S3.

### 3.1 Manipulation Checks

**FIGURE 2:**
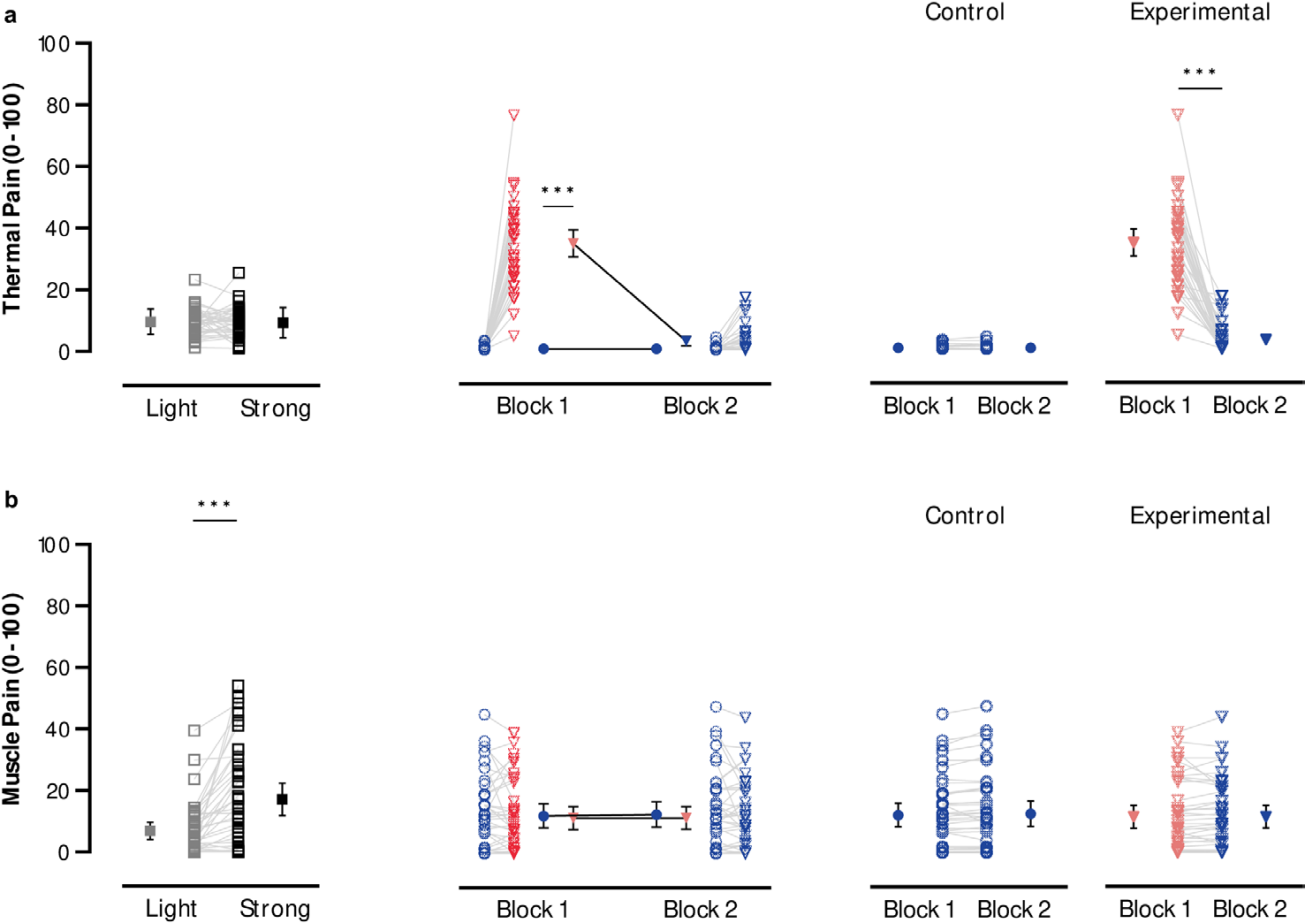
Changes in thermal pain (a) and muscle pain (b) ratings between light (grey squares) and strong (black squares) fixed perceived effort intensities as well as condition × block effects in the same measures between the control (circles) and experimental (triangles) at Block 1 and Block 2. Red triangles indicate when the painful stimulation was applied in the experimental condition whereas blue triangles and circles indicate when the warm control stimulation was applied in the control and experimental conditions. Grey lines represent comparisons of the same participant's data across different intensities and condition × block. Colour filled icons represent group mean data with error bars depicting 95% confidence intervals. One symbol (*) represents p<0.05, two symbols represent p<0.01, and three symbols represent p<0.001. Denotation of ns represents non‐significant findings.

All force and EMG parameters were higher within the strong than the light fixed perceived effort intensity (see Table S3 for all statistical details). Muscle pain ratings were higher during the strong versus light intensity. Pairwise comparisons of experimental Block 1 and control Block 1 confirm that thermal pain ratings were higher during pain versus control conditions $\left(t_{39}=-15.820,p<0.001,d=-3.149\left[-4.284,-2.014\right]\right)$. Pairwise comparisons also showed that thermal pain ratings were higher in experimental Block 1 versus the within‐trial control during experimental Block 2 $\left(t_{39}=13.535,p<0.001,d=2.918\left[1.821,4.015\right]\right)$. Although a warm control stimulation was applied in Block 2 of both conditions, pairwise comparisons showed higher thermal pain ratings in the experimental condition $\left(t_{39}=-3.572,p=0.002,d=-0.231\left[-0.425,-0.037\right]\right)$. Eleven participants rated pain above zero during experimental Block 2 (mean ± SD rating: 6.9 ± 5.3). Mean ratings corresponded to a descriptor of ‘very light’ and suggest a residual pain effect of thermal stimulation from Block 1 (Figure 2a). Muscle pain was not affected by the pain conditions; thus, participants appropriately distinguished between muscle (dominant arm) and thermal (non‐dominant arm) pain (Figure 2b).

### 3.2 Effects of Thermal Pain Stimulation

**FIGURE 3:**
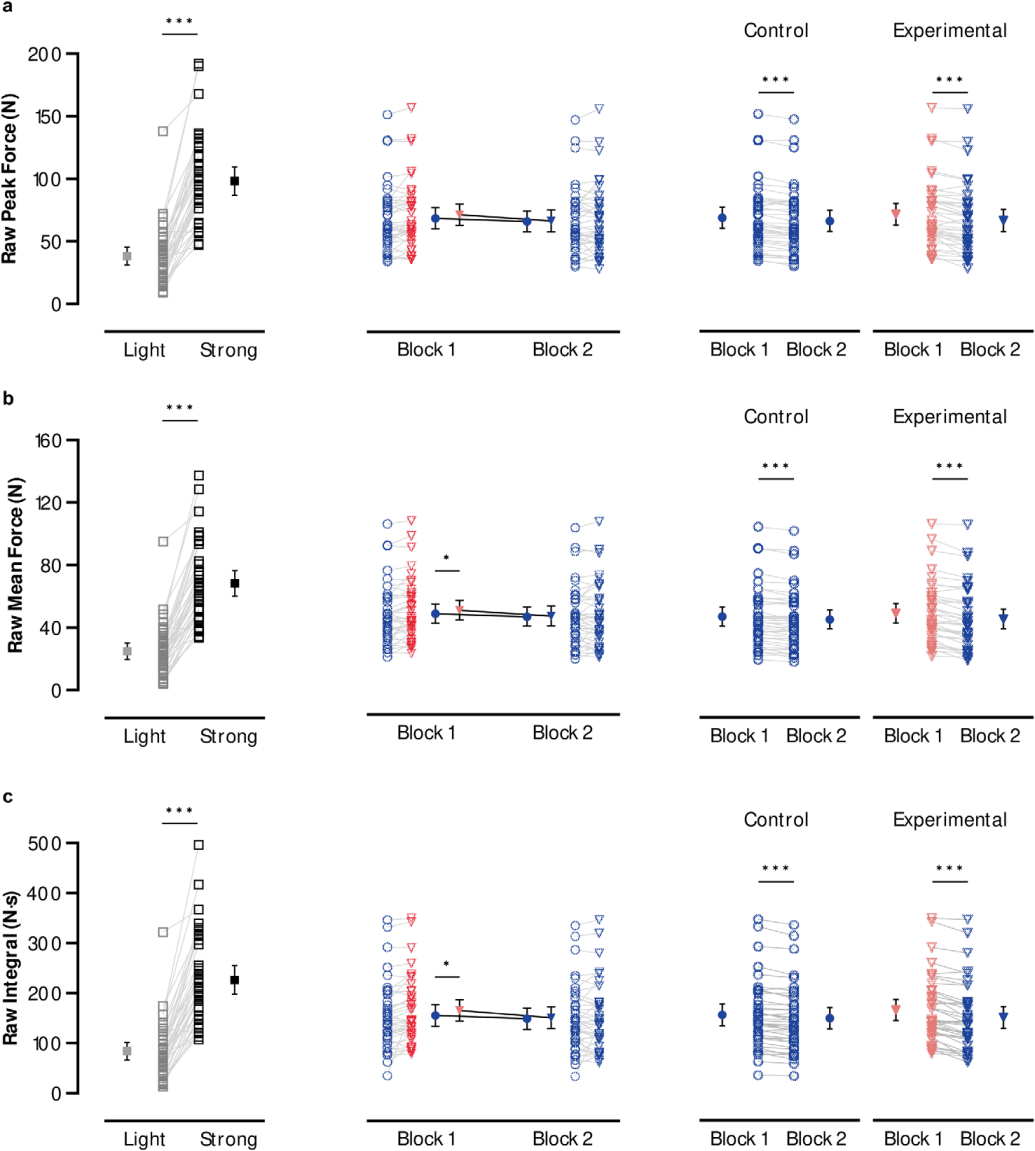
Changes in raw peak force (a), mean force (b), and force‐time integral (c) between light (grey squares) and strong (black squares) fixed perceived effort intensities as well as condition × block effects in the same measures between the control (circles) and experimental (triangles) at Block 1 and Block 2. Red triangles indicate when the painful stimulation was applied in the experimental condition whereas blue triangles and circles indicate when the warm control stimulation was applied in the control and experimental conditions. Grey lines represent comparisons of the same participant's data across different intensities and condition × block. Colour filled icons represent group mean data with error bars depicting 95% confidence intervals. One symbol (*) represents p<0.05, two symbols represent p<0.01, and three symbols represent p<0.001. Denotation of ns represents non‐significant findings.

All raw force parameters showed large condition × block effects (see Table S3b). Between‐condition comparisons (experimental Block 1 vs. control Block 1) showed peak force was higher in the painful versus control condition $\left(t_{39}=-2.289,p=0.055,d=0.091\left[-0.220,0.402\right]\right)$ but this only trended near significance (Figure 3a). Mean force $\left(t_{39}=-2.442,p=0.038,d=-0.098\left[-0.214,0.018\right]\right)$, and force‐time integral $\left(t_{39}=-2.466,p=0.036,d=-0.127\left[-0.275,0.022\right]\right)$ were higher during painful versus control conditions (Figure 3b,c). Peak RFD did not differ between during painful and control conditions $\left(t_{39}=-1.951,p=0.117,d=0.081\left[-0.037,0.199\right]\right)$ (Figure S2). Normalised peak force and peak RFD did not differ between painful and control conditions $\left(p^{\prime }s>0.367-0.437,d^{\prime }s<0.076\right)$. In the experimental condition, all force parameters were higher at Block 1 compared to Block 2 $\left(p^{\prime }s<0.001,d^{\prime }s=0.093-0.168\right)$ however, this comparison may be compounded by time‐on‐task‐related fatigue (Figure S3).

**FIGURE 4:**
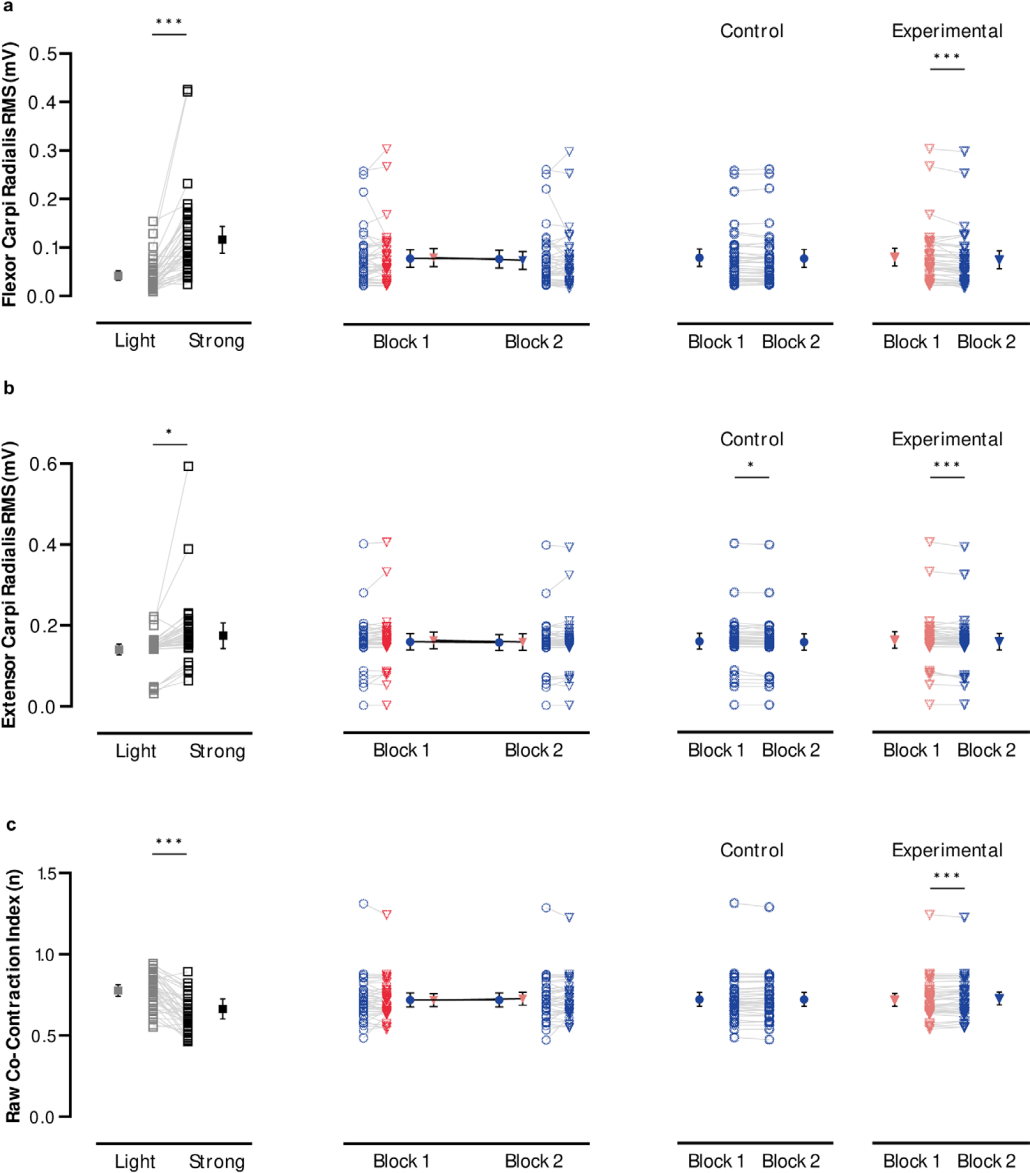
Changes in raw flexor carpi radialis (a) and extensor carpi radialis root mean squared activity (b), as well as raw co‐contraction index (c) between light (grey squares) and strong (black squares) fixed perceived effort intensities as well as condition × block effects in the same measures between the control (circles) and experimental (triangles) at Block 1 and Block 2. Red triangles indicate when the painful stimulation was applied in the experimental condition whereas blue triangles and circles indicate when the warm control stimulation was applied in the control and experimental conditions. Grey lines represent comparisons of the same participant's data across different intensities and condition × block. Colour filled icons represent group mean data with error bars depicting 95% confidence intervals. One symbol (*) represents p<0.05, two symbols represent p<0.01, and three symbols represent p<0.001. Denotation of ns represents non‐significant findings.

All EMG measures showed moderate‐large condition × block effects (Table S3c). Flexor carpi radialis RMS and co‐contraction index did not differ between experimental Block 1 and control Block 1 ($p^{\prime }s<0.662,d=0.007-0.023$; Figure 4a–c). Extensor carpi radialis RMS was higher in experimental Block 1 than control Block 1 $\left(t_{39}=-1.992,p=0.053,d=0.046\left[-0.264,0.356\right]\right)$ but this only trended near significance. Normalised flexor and extensor carpi radialis RMS and normalised co‐contraction index did not differ between experimental and control Block 1 $\left(p^{\prime }s<0.187,d^{\prime }s>0.161\right)$. In the experimental condition, flexor $\left(t_{39}=3.952,p<0.001,d=0.082\left[0.019,0.146\right]\right)$ and extensor $\left(t_{39}=4.076,p<0.001,d=0.054\left[0.013,0.094\right]\right)$ carpi radialis RMS, and co‐contraction index were higher during Block 1 than Block 2. Co‐contraction index was higher during Block 2 than Block 1 of the experimental condition $\left(t_{39}=-4.074,p<0.001,d=0.057\left[-0.014,0.100\right]\right)$. In the experimental condition, normalised flexor $\left(t_{39}=3.693,p=0.001,d=0.150\left[0.028,0.253\right]\right)$ and extensor $\left(t_{39}=3.934,p<0.001,d=0.054\left[0.012,0.096\right]\right)$ carpi radialis RMS were higher in Block 1 than Block 2 (Figure S4). Normalised co‐contraction index was higher in Block 2 than Block 1 of the experimental condition $\left(t_{39}=-4.520,p<0.001,d=0.077\left[0.024,0.131\right]\right)$.

### 3.3 Effects of Naturally Occurring Muscle Pain

#### 3.3.1 Effects of Muscle Pain With Intensity and Sex as Covariates

The first regression analysis revealed that muscle pain ratings had a positive effect (i.e., increase in force production) on peak force, mean force, and force‐time integral $\left(p^{\prime }s<0.001\right)$. However, when adding intensity as a factor, for each one‐unit increase in muscle pain ratings, peak force *decreased* by 0.45 N, mean force by 0.32 N, and force‐time integral by 1.02 N·s. (Tables S4–S6, Figure S5). The interaction between intensity and muscle pain approached significance for force‐time integral $\left(p=0.052\right)$ and mean force $\left(p=0.063\right)$. The interaction between intensity and muscle pain was significant for peak force $\left(p=0.040\right)$. Force decreases were more pronounced in the light versus strong intensity for all force measures (Figure S6) and the effect of muscle pain remained significant $\left(p<0.001\right)$. Exploratory analysis showed no difference in thermal pain ratings between sexes $\left(U=308167,p=0.130,r=0.037\right)$. Muscle pain ratings were higher amongst females that males $\left(U=290372,p=0.001.r=0.093\right)$ as females were more likely to rate some muscle pain versus males who often reported muscle pain as 0 ‘no pain’ (Table S7, Figure S7). Males exerted a higher peak force $\left(U=300802,p=0.038.r=0.060\right)$, mean force $\left(U=295151,p=0.007.r=0.077\right)$, and force‐time integral $\left(U=286644,p<0.001.r=0.104\right)$ than females. The third model of the first linear regression analysis showed that force measures decreased more rapidly for males compared to females as muscle pain increased (Figure S8), though no muscle pain × intensity × sex interaction was observed $\left(p^{\prime }s>0.321\right)$, the effect of muscle pain remained significant $\left(p<0.001\right)$.

#### 3.3.2 Effects of Muscle Pain With Objective and Subjective Fatigue as Covariates

To integrate objective (MVC) and subjective (VAS) manifestations of fatigue, we computed mean values per participant and per condition. Based on these averages, we first conducted a linear regression model to examine the effect of muscle pain on peak force, mean force, and force‐time integral. Similar to the first analysis, the model revealed a marginal effect of muscle pain on force‐time integral $\left(p=0.075\right)$, suggesting that higher muscle pain was associated with increased force production. However, when adding intensity (light vs. strong) as a predictor, interaction effects were not significant $\left(p^{\prime }s>0.374\right)$. Main effects of muscle pain were approached significance $\left(p=0.076\right)$ but the direction of effect was reversed (i.e., as muscle pain increased, force production decreased). This was identical to the first regression analysis and suggests that the initially observed positive relationship between pain and force production was confounded by intensity, as both force output and muscle pain are higher in the strong intensity.

In a third model, muscle fatigue was controlled by including the changes in peak force from pre‐post condition MVCs, Subjective fatigue was also controlled by included changes in pre‐post condition VAS ratings of fatigue. With these additional covariates, the effect of muscle pain was statistically significant $\left(p^{\prime }s=0.029-0.038\right)$ but the interactions were not significant $\left(p^{\prime }s>0.205\right)$. Specifically, force‐time integral decreased by 1.84 N·s as muscle pain ratings increased by one unit (Figure S9). We also observed a significant negative effect of muscular fatigue on force‐time integral $\left(\beta =-0.96,p<0.001\right)$. Subjective fatigue did not affect force production $\left(p^{\prime }s>0.390\right)$.

### 3.4 Intensity‐Related Effects and Interactions

Intensity main effects were observed for all EMG parameters. Flexor $\left(t_{39}=-6.898,p<0.001,d=1.075\left[0.675,1.475\right]\right)$ and extensor $\left(t_{39}=-2.565,p=0.014,d=0.444\left[0.079,0.808\right]\right)$ carpi radialis RMS was lower in the light versus strong condition. Normalised flexor $\left(t_{39}=-17.144,p<0.001,d=1.290\left[0.870,1.709\right]\right)$ and extensor $\left(t_{39}=-11.010,p<0.001,d=0.553\left[0.248,0.858\right]\right)$ carpi radialis RMS was lower in light than strong condition. Raw $\left(t_{39}=4.128,p<0.001,d=0.713\left[0.327,1.098\right]\right)$ and normalised $\left(t_{39}=6.140,p<0.001,d=0.857\left[0.513,1.201\right]\right)$ co‐contraction index was higher in the light versus strong condition (Figure 4).

When assessing whether the perceived effort intensity impacted the thermal pain stimulation effect (intensity × condition × block interaction) no significant effects were found for any dependent variable.

### 3.5 Maximal Voluntary Contraction Changes

Peak force $\left(F_{1,39}=94.234,p<0.001,\eta_{p}^{2}=0.707\left[0.528,0.794\right]\right)$, mean force $\left(F_{1,39}=100.391,p<0.001,\eta_{p}^{2}=0.720\left[0.547,0.804\right]\right)$, and force − time integral $\left(F_{1,39}=53.182,p<0.001,\eta_{p}^{2}=0.577,\left[0.352,0.701\right]\right)$ during MVCs all showed main effects of time with large effect sizes. Peak force $\left(t_{39}=-9.707,p<0.001,d=0.231\left[0.160,0.303\right]\right)$, mean force $\left(t_{39}=-10.020,p<0.001,d=0.235\;\left[0.163,0.306\right]\right)$, and force‐time integral $\left(t_{39}=7.293,p<0.001,d=0.225\left[0.144,0.305\right]\right)$ all decreased from pre‐ to post‐condition. There was no time‐related main effect for Peak RFD during MVCs $\left(F_{1,39}=3.731,p=0.061,\eta_{p}^{2}=0.087\left[0.000,0.283\right]\right)$. No main effects of intensity were observed for intensity across all force measures during MVCs $\left(p^{\prime }s<0.080\right)$. Peak force $\left(F_{1,39}=14.672,p<0.001,\eta_{p}^{2}=0.273\left[0.064,0.463\right]\right)$, mean force $\left(F_{1,39}=13.280,p<0.001,\eta_{p}^{2}=0.254\left[0.052,0.446\right]\right)$, force − time integral $\left(F_{1,39}=4.591,p=0.038,\eta_{p}^{2}=0.105\left[0.000,0.446\right]\right)$, and peak RFD $\left(F_{1,39}=4.127,p=0.049,\eta_{p}^{2}=0.096\left[0.000,0.284\right]\right)$ measures during MVCs all exhibited main intensity × time interactions. Over the course of each fixed perceived effort task, the strong intensity invoked a greater time‐based fatigue effect compared to the light intensity. Conditions (control vs. experimental) showed no effect on any MVC‐related changes $\left(p^{\prime }s>0.396\right)$. Mean differences between pre‐ to post‐condition was 23.12 N for peak force, 20.05 N for mean force, and 104.05 N·s for force‐time integral (Figure S10).

### 3.6 Motivation, Boredom, and Fatigue

Repeated measures ANOVAs showed decreases in motivation over time $\left(F_{1,39}=6.666,p=0.008,\eta_{p}^{2}=0.146,\left[0.006,0.341\right]\right)$. Repeated measures ANOVAs showed increases in boredom $\left(F_{1,39}=10.097,p<0.001,\eta_{p}^{2}=0.206\left[0.028,0.401\right]\right)$ and perceived fatigue $\left(F_{1,39}=14.686,p<0.001,\eta_{p}^{2}=0.274\left[0.064,0.463\right]\right)$ over time (Figure S11). Bonferroni comparisons showed motivation ratings only differed between mid‐trial and post‐trial ratings $\left(t_{39}=3.513,p=0.003,d=0.279\left[0.065,0.492\right]\right)$. Pairwise comparisons showed boredom ratings differed from pre‐ to mid‐trial $\left(t_{39}=-2.936,p=0.017,d=0.335\left[0.034,0.635\right]\right)$ and pre‐ to post‐trial $\left(t_{39}=-4.360,p<0.001,d=-0.485\left[-0.796,-0.175\right]\right)$. Pairwise comparisons showed fatigue ratings differed from pre‐ to post‐trial $\left(t_{39}=-4.250,p<0.001,d=-0.347\left[-0.574,-0.120\right]\right)$ and mid to post‐trial $\left(t_{39}=-4.116,p<0.001,d=-0.227\left[-0.379,-0.075\right]\right)$. No intensity main effects were observed for VAS motivation, boredom, and fatigue ratings $\left(p^{\prime }s>0.245\right)$. No intensity × time interactions were observed suggesting fixed perceived effort intensity did not impact the time‐based changes in VAS reports.

### 3.7 Exploratory Analysis

Exploratory analysis relating to additional dependent variables (e.g., false starts, reaction times, contraction duration), condition × block × repetition, and intensity × condition × block × repetition interactions, and exercise‐induced hypoalgesia effects are available in the Supporting Information (Figures S12–S22, Materials S10–S17).

## 4 Discussion

This study aimed to investigate the effect of experimentally induced contralateral thermal pain and naturally occurring muscle pain on force production during an isometric handgrip task performed at a fixed perceived effort. Contrary to our first hypotheses, force production was higher during contralateral thermal pain than in the control. Consistent with our second hypothesis, when naturally occurring muscle pain increased, force production decreased. Together, these results suggest that force production changes during a self‐regulated task are pain‐type dependent.

During the fixed perceived effort task, force production and associated muscle activation were higher than at a strong versus a light effort. Participants also reported more muscle pain when exercising at a strong compared to a light effort. Finally, participants reported a higher pain in Block 1 of the experimental compared to the control condition. These results confirm that our experimental manipulations were successful.

Most of the literature on the effect of pain on task performance and force production has used fixed workload tasks (e.g., Aboodarda et al. 2020; Norbury et al. 2022a). These studies suggest that when the task is within the individual's capacity, pain could increase the effort perceived to maintain task demands, compared to a control condition (Aboodarda et al. 2020; Canestri et al. 2019; Norbury et al. 2022a). In contrast, our study involved a task where participants could freely regulate their force production, based on their perceived effort intensity. Subsequently, participants could self‐regulate their force, thus offering a unique assessment of the effects of pain on force production. Contrary to our hypothesis, we observed a higher force production in the presence of contralateral thermal pain versus a warm control stimulation.

The stimulation intensities were individualised to induce a strong pain, below the participant's maximal tolerance. Therefore, following the familiarisation visit, participants knew they would be able to tolerate this pain intensity throughout each experimental session without requiring the removal of the thermode from their skin. We speculate that in this specific context, a small increase in force production may be a convenient adaptive response to direct attention away from the thermal pain experience (Torta et al. 2017). Although some studies contend that force should decrease when faced with any pain type for a given workload (e.g., Chowdhury et al. 2022; Wender et al. 2023), we propose that, as a protective measure, reduced force during our task would be counterproductive as it could likely draw more immediate attention back to thermal pain (Torta et al. 2017; Van Damme et al. 2010).

To help explain the force production results identified in our a priori analyses, we conducted an inductive exploratory analysis (Briand et al. 2025) of contraction duration across conditions, as well as thermal pain intensity ratings at rest and during the motor task. Although the exploratory findings on contraction duration should be interpreted with caution because the fixed delay between trials may have introduced anticipatory effects, the analyses suggested longer contraction durations during painful versus warm stimulations. Previous studies have observed reduced pain intensity during cognitively demanding tasks (Tabry et al. 2020), and physical tasks are well known to induce exercise‐related hypoalgesia (Naugle et al. 2012). Therefore, we speculate that when participants are free to self‐regulate their force production while maintaining a fixed level of perceived effort, increasing effort (i.e., allocating greater resources to the task) may be a way to promote an effort‐induced hypoalgesia response (Naugle et al. 2012; Tabry et al. 2020). A potential hypoalgesia response may result from the diversion of attention away from the integration of nociceptive signals (Torta et al. 2017). However, we did not observe lower thermal pain ratings with increasing effort intensity, which challenges the idea that more intense exercise would produce greater distraction from thermal sensations and consequently reduce thermal pain. As investigating hypoalgesia was not a primary aim of this study, we did not include a direct measure of attention. Therefore, given the absence of such a measure and the exploratory nature of these analyses, future studies should specifically investigate the relationship between effort intensity and hypoalgesia effects while incorporating measures of attention.

We also investigated the effects of naturally occurring muscle pain on force production. Interestingly, both regression analyses identified that when controlling for the intensity of the task, as muscle pain ratings increased, force production decreased—a contrasting effect to experimental contralateral thermal pain. To strengthen this finding, when objective and subjective measures of fatigue were added as covariates to a similar regression analysis, force production still decreased as muscle pain increased. Naturally occurring muscle pain involves distinct pathways that culminate in an altogether different pain experience compared to thermal pain (Graven‐Nielsen 2006). Consequently, individuals experiencing naturally occurring muscle pain tend to evaluate it as non‐damaging (O'Connor and Cook 1999; O'Malley et al. 2024) and are aware that it draws directly from task engagement (Cook et al. 1997). Therefore, in the context of our study, an increase in handgrip force production would likely be counterproductive as this would only promulgate further metabolite build‐up in the forearm muscles (Aboodarda et al. 2020), leading to greater nociceptive stimulation (Pollak et al. 2014), and subsequently increased muscle pain intensity (Cook et al. 1997; O'Connor and Cook 1999).

Collectively, our results suggest that the effects of pain on self‐regulated force production are likely pain‐type dependent, an observation that may have been overlooked in previous studies because their experimental designs typically relied on fixed‐workload tasks. Such foundational studies have primarily examined the neurophysiological and biomechanical adaptations that support maintenance of a target force, highlighting changes in force variability (e.g., Arvanitidis et al. 2025), load sharing (e.g., Hug et al. 2014), and intra‐muscular coordination (e.g., Falla et al. 2017). Alternatively, fixed perceived effort tasks allow individuals to self‐regulate force production while maintaining a constant effort intensity (O'Malley et al. 2023). As effort and its perception are proposed to play a central role in the regulation human behaviour, fixed perceived effort may better reflect everyday responses to pain (Arendt‐Nielsen and Graven‐Nielsen 2008; Cook et al. 1997). Therefore, by using a novel fixed perceived‐effort paradigm, our findings extend current understanding of how pain influences force production. We encourage future research to use fixed perceived effort tasks to investigate the effect of different pain types on self‐regulated force production.

There are several strengths and limitations of our study that should be considered in future research. Using a naturally occurring muscle pain model in healthy individuals may help bridge the gap between experimental and clinical pain research. This would align with recent research, such as movement‐evoked electrical pain in the lumbar region (Devecchi et al. 2025), aiming at developing and using experimental pain models with improved ecological relevance, to facilitate transfer from the lab to the field. However, eliciting naturally occurring muscle pain requires exercise, which also generates numerous sensations and perceptions that make it difficult to isolate the specific effects of muscle pain on force production (Graven‐Nielsen 2006; O'Malley et al. 2024). Therefore, many studies have used experimental pain models, like thermal stimulations (e.g., Billot et al. 2018), which provide greater experimental control and standardisation and are often used as proxies for naturally occurring muscle pain. Nevertheless, experimental and naturally occurring muscle pain differ in their neurophysiological mechanisms (Almeida et al. 2004), cortical processing (Hofbauer et al. 2001), and cognitive evaluations (O'Malley et al. 2024). Unlike experimental thermal pain, naturally occurring muscle pain can be directly modulated by the individual through changes in force production (Arendt‐Nielsen and Graven‐Nielsen 2008; O'Connor and Cook 1999). Accordingly, experimental and naturally occurring muscle pain are likely to have different effects on force production (O'Malley et al. 2024), like we observed. It is also well‐established that different experimental pain models induce distinct neurophysiological and biomechanical adaptations sustaining performance, including differences in force steadiness (Arvanitidis et al. 2025), muscle activation, and subsequent movement kinetics (Bank et al. 2013). Future research should compare the effects of naturally occurring muscle pain with those of experimental pain models other than thermal pain to further understand how different types of pain influence force production.

Another consideration is that pain location may influence changes in force production. In this study, thermal pain was applied to the contralateral, non‐exercising arm, whereas muscle pain was experienced in the exercising arm. Applying experimental thermal pain to the exercising arm (ipsilateral pain) may produce different effects on force production than those observed here. Some studies suggest that contralateral experimental pain has a smaller impact on force production than ipsilateral pain (Azevedo et al. 2022; Norbury et al. 2022b). Additionally, Zhang et al. (2025) recently reported differences in cortical inhibition depending on whether experimental pain was applied to the contralateral or ipsilateral limb. Therefore, future research should investigate the influence of pain location using a similar design to that employed in the present study.

A further consideration relates to fatigue. As fatigue is known to disrupt the ability of humans to dissociate perceptions of effort from force (Jones and Hunter 1983; Pageaux 2016), we cannot exclude the possibility that, as fatigue developed over time‐on‐task, individuals progressively relied not only on effort perception but also on force‐related sensations when regulating their performance. Consequently, our findings may have been influenced by the emergence of a perceptual drift over the course of the task, a phenomenon proposed to occur in the presence and absence of visual feedback (Limonta et al. 2015; Pawłowski et al. 2024). Next, while our results suggest that the intensity of the physical task does not interact with thermal pain‐related effects on force production, crucially our handgrip task involved a small muscle mass. It is well known that increasing the muscle mass involved in a physical task increases the cardiorespiratory responses to the task (MacInnis et al. 2017). Consequently, as increased cardiorespiratory responses to the task are associated with negative affective responses and aversive perceptions of effort and pain (Ekkekakis et al. 2011), future studies should conceptually replicate our results with physical tasks involving larger muscle groups (e.g., knee extensions, functional lower body movements like squatting, or cycling).

A last suggestion for future research relates to sex‐based differences. When conducting exploratory analysis by including sex as a factor in our regression analysis, females maintained force production, whereas males decreased force outputs as muscle pain increased. Thus, we encourage future studies to use an a priori approach to investigate the differences in force production due to naturally occurring muscle pain between sexes (e.g., Cook et al. 1998).

## 5 Conclusions

By using a motor task where participants were free to regulate their force production, this study observed a pain‐type effect on force production. In the presence of thermal pain, participants increased their force to maintain the same level of perceived effort. On the contrary, increased naturally occurring muscle pain was associated with a decrease in force production. The intensity of the task did not interact with the effects of thermal pain on force production. Our regression analysis implies that muscle pain remains a strong predictor of force decreases despite fatigue manifestations. As fatigue and naturally occurring muscle pain often develop concomitantly during physical exercise, the completion of physical tasks where the participants are free to regulate their motor output based on a fixed perceived effort could provide unique opportunities for better understanding the interaction between pain and fatigue. We encourage future studies to dissociate between pain types and other phenomena rooted within physical exercise (e.g., fatigue) when investigating the complex relation between pain and force production.

## Author Contributions

C.A.O'M. – conceptualisation, data curation, formal analysis, funding acquisition, investigation, methodology, project administration, validation, visualisation, writing – original draft, writing – review and editing. T.M. – conceptualisation, data curation, formal analysis, investigation, methodology, validation, writing – review and editing. M.B. – conceptualisation, data curation, investigation, methodology, writing – review and editing. I.M. – conceptualisation, methodology, data curation, investigation. C.L.F. – conceptualisation, funding acquisition, supervision. A.R.M. – conceptualisation, funding acquisition, supervision. P.R. – conceptualisation, data curation, methodology, validation. B.P. – conceptualisation, formal analysis, funding acquisition, investigation, methodology, project administration, supervision, validation, visualisation, writing – review and editing.

## Funding

CAO'M was a recipient of the Mitacs Canada Global Fellowship (Funding Number: IT32394), Canada‐UK Foundation Travel Award (no funding number available), and University of Kent Erasmus+/Turing Scheme (no funding number available) which supported the current project. CAO'M is currently a researcher of an industry funded project between the University of Exeter and Smiths Detection Limited (Project Number: 1826975). TM is supported by the Fonds de Recherche du Québec—Nature et Technologies (FRQNT), Postdoctoral scholarship. MB is supported by a doctoral postgraduate scholarship from the National Sciences and Engineering Research Council (NSERC) of Canada and a doctoral scholarship from the FRQNT. IM is supported by the FRQNT doctoral scholarship. BP is supported by the NSERC (Funding Number: RGPIN‐2019‐05057) and the Chercheur Boursier Junior 1 award from the Fonds de Recherche du Québec—Santé.

## Disclosure

Declarations: For the purpose of open access, the authors have applied a Creative Common Attribution (CC BY) licence to any preprint and author accepted manuscript version arising from this submission. In the revisions, the authors used ChatGPT plus to assist with language editing. The authors reviewed and edited the manuscript content as needed and take full responsibility for the content of the published article. All scientific content, data interpretation, and conclusions were generated solely by the authors.

## Ethics Statement

Ethical permission for this study was granted by the Comité d'Ethique de la Recherche Clinique at the Université de Montréal and CRIUGM under project number: CER VN‐22‐23‐16 on October 18th, 2022. All participants provided written informed consent prior to any participation in the study and all procedures were conducted in accordance with the Declaration of Helsinki.

## Conflicts of Interest

The authors declare no conflicts of interest.

## Supporting information


**Figure S1:** Sensitivity analysis for statistical outputs.
**Figure S2:** Raw peak RFD intensity, condition × block effects.
**Figure S3:** Normalised peak force and peak RFD intensity and condition × block effects.
**Figure S4:** Normalised EMG intensity and condition × block effects.
**Figure S5:** Linear regression of force related changes according to muscle pain rating changes.
**Figure S6:** Linear regression of force related changes according to muscle pain rating changes and intensity.
**Figure S7:** Pairwise comparisons in pain rating by participant sex.
**Figure S8:** Linear regression of force related changes according to muscle pain rating changes, intensity, and participant sex.
**Figure S9:** Linear regression of force production changes according to muscle pain ratings, intensity, and fatigue measures.
**Figure S10:** Pre‐ to post‐trial changes in MVC force measures.
**Figure S11:** Pre‐, mid‐ and post‐trial changes in perceived VAS responses.
**Figure S12:** False starts to contract and relax prompt intensity and condition × block effects.
**Figure S13:** Contract and relax reaction times and contraction duration intensity and condition × block effects.
**Figure S14:** Heart rate and breathing frequency intensity and condition × block effects.
**Figure S15:** Block 1 pain rating changes per repetition.
**Figure S16:** Block 1 raw force changes per repetition.
**Figure S17:** Block 1 normalised force changes per repetition.
**Figure S18:** Block 1 raw EMG changes per repetition.
**Figure S19:** Block 1 normalised EMG changes per repetition.
**Figure S20:** Block 1 false starts, response time and contraction duration changes per repetition.
**Figure S21:** Block 1 heart rate and breathing frequency changes per repetition.
**Figure S22:** Changes in thermal pain ratings over each time‐point.
**Table S1:** Overview of participant characteristics.
**Table S2:** Overview of randomisation and counterbalance orders.
**Table S3:** Statistical outputs for main and interaction effects.
**Table S4:** Peak force regression analysis 1 output.
**Table S5:** Mean force regression analysis 1 output.
**Table S6:** Force‐time integral regression analysis 1 output.
**Table S7:** Student t test output for pain ratings by participant sex.
**Table S8:** Peak force regression analysis 2 output.
**Table S9:** Mean force regression analysis 2 output.
**Table S10:** Force‐time integral regression analysis 2 output.
**Material S1:** Dijon physical activity score.
**Material S2:** Edinburgh handedness questionnaire.
**Material S3:** 15‐item task specific motivation.
**Material S4:** 13‐item pain catastrophising scale.
**Material S5:** 40‐item five facets of mindfulness questionnaire.
**Material S6:** Borg CR100 scale.
**Material S7:** Pre‐trial motivation, boredom and fatigue VAS scales.
**Material S8:** Midtrial motivation, boredom and fatigue VAS scales.
**Material S9:** Post‐trial motivation, boredom and fatigue VAS scales.
**Material S10:** Intensity‐related effects on psychological parameters, heart rate and breathing frequency.
**Material S11:** Influence of time‐on‐task on pain stimulation responses (Condition × Block × Repetition Interactions).
**Material S12:** Other exploration of interaction effects.
**Material S13:** Exercise‐induced hypoalgesia.
**Material S14:** Bonferroni‐Holm correction tables for condition × block comparisons of all dependent variables.
**Material S15:** Bonferroni‐Holm correction tables for repetition comparisons of all dependent variables.
**Material S16:** Bonferroni‐Holm correction tables for intensity × condition × block comparisons of all dependent variables.
**Material S17:** Bonferroni‐Holm correction files for condition × block × repetition comparison.

## Data Availability

This study was preregistered at https://osf.io/8tbm3/. Supporting Informations and preprint version(s) are available at the OSF link provided. All individual participant data is presented in figures and Supporting Information. In accordance with the policies of the institution which awarded ethical approval, anonymised data cannot be uploaded to the open science framework. Data can be requested from the corresponding author(s) who will log a request with the institutional ethics board to share the data. Any additional questions can also be directed towards the corresponding author(s).
