# Supplementary material for: Contralateral Thermal Pain and Naturally Occurring Muscle Pain Have Different Effects on Force Production During a Fixed Perceived Effort Handgrip Task
Source: Eur J Pain. 2026 Jul 8;30(6):e70329. doi: 10.1002/ejp.70329 (PMC13344111; doi:10.1002/ejp.70329)
Supplement: Supplementary file 1 — Figure S1: Sensitivity analysis for statistical outputs. Figure S2: Raw peak RFD intensity, condition × block effects. Figure S3: Normalised peak force and peak RFD intensity and condition × block effects. Figure S4: Normalised EMG intensity and condition × block effects. Figure S5: Linear regression of force related changes according to muscle pain rating changes. Figure S6: Linear regression of force related changes according to muscle pain rating changes and intensity. Figure S7: Pairwise comparisons in pain rating by participant sex. Figure S8: Linear regression of force related changes according to muscle pain rating changes, intensity, and participant sex. Figure S9: Linear regression of force production changes according to muscle pain ratings, intensity, and fatigue measures. Figure S10: Pre‐ to post‐trial changes in MVC force measures. Figure S11: Pre‐, mid‐ and post‐trial changes in perceived VAS responses. Figure S12: False starts to contract and relax prompt intensity and condition × block effects. Figure S13: Contract and relax reaction times and contraction duration intensity and condition × block effects. Figure S14: Heart rate and breathing frequency intensity and condition × block effects. Figure S15: Block 1 pain rating changes per repetition. Figure S16: Block 1 raw force changes per repetition. Figure S17: Block 1 normalised force changes per repetition. Figure S18: Block 1 raw EMG changes per repetition. Figure S19: Block 1 normalised EMG changes per repetition. Figure S20: Block 1 false starts, response time and contraction duration changes per repetition. Figure S21: Block 1 heart rate and breathing frequency changes per repetition. Figure S22: Changes in thermal pain ratings over each time‐point. Table S1: Overview of participant characteristics. Table S2: Overview of randomisation and counterbalance orders. Table S3: Statistical outputs for main and interaction effects. Table S4: Peak force regression analysis 1 output. Table S5: Mean force [file EJP-30-0-s001.docx]

Supplementary Figures, Tables, and Materials File for

Experimental thermal pain and naturally occurring muscle pain have different effects on force production during a fixed perceived effort handgrip task.

Callum A. O’Malley^1,2,3🞷^[^ID^](https://orcid.org/0000-0001-6685-5800), Thomas Mangin^2,3,4^[^ID^](https://orcid.org/0000-0002-3393-7946), Maxime Bergevin^2,3,4^[^ID^](https://orcid.org/0000-0003-2933-1578), Ilaria Monti^2,3,4^[^ID^](https://orcid.org/0000-0002-3731-2361), Christopher L. Fullerton^5^[^ID^](https://orcid.org/0000-0002-6902-7960), Alexis R. Mauger^6^[^ID^](https://orcid.org/0000-0001-9302-5183), Pierre Rainville^2,7^[^ID^](https://orcid.org/0000-0001-9801-757X), & Benjamin Pageaux^2,3,4^[^ID^](https://orcid.org/0000-0002-0343-8900).

^1^ Department of Public Health and Sport Sciences, University of Exeter, Exeter, United Kingdom, EX1 2LU.

^2^ Centre de Recherche de l’Institut Universitaire de Gériatrie de Montréal (CRIUGM), Montréal, Canada, H3W 1W4.

^3^ École de Kinésiologie et des Sciences de l’Activité Physique (EKSAP), Faculté de Médecine, Université de Montréal, Montréal, Canada, H3T 1J4.

^4^ Centre Interdisciplinaire de Recherche sur le Cerveau (CIRCA), Montréal, Canada, H3T 1P1.

^5^ Faculty of Health Sciences and Sport, University of Stirling, Stirling, United Kingdom, FK9 4LA

^6^ School of Natural Sciences, University of Kent, Canterbury, United Kingdom, CT2 7PE.

^7^ Département de Stomatologie, Faculté de Médecine Dentaire, Université de Montréal, Montréal, Canada, H3T IJ4.

^🞷^ Corresponding Author

Corresponding Author Contact Details

Mailing Address: Dr Callum A. O’Malley, Haighton Building, St Luke’s Campus, Exeter, Devon, United Kingdom, EX1 2LU.

Email Address: c.omalley@exeter.ac.uk

Institutional URL: https://medicine.exeter.ac.uk/phss/


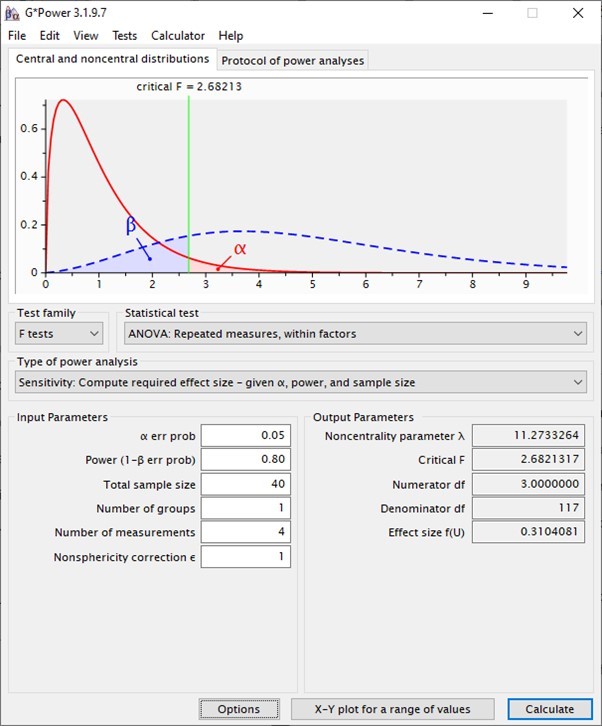


Figure S1. Sensitivity analysis for participation sample size justification from G*Power (Version 3.1.9.7: Düsseldorf, Germany)


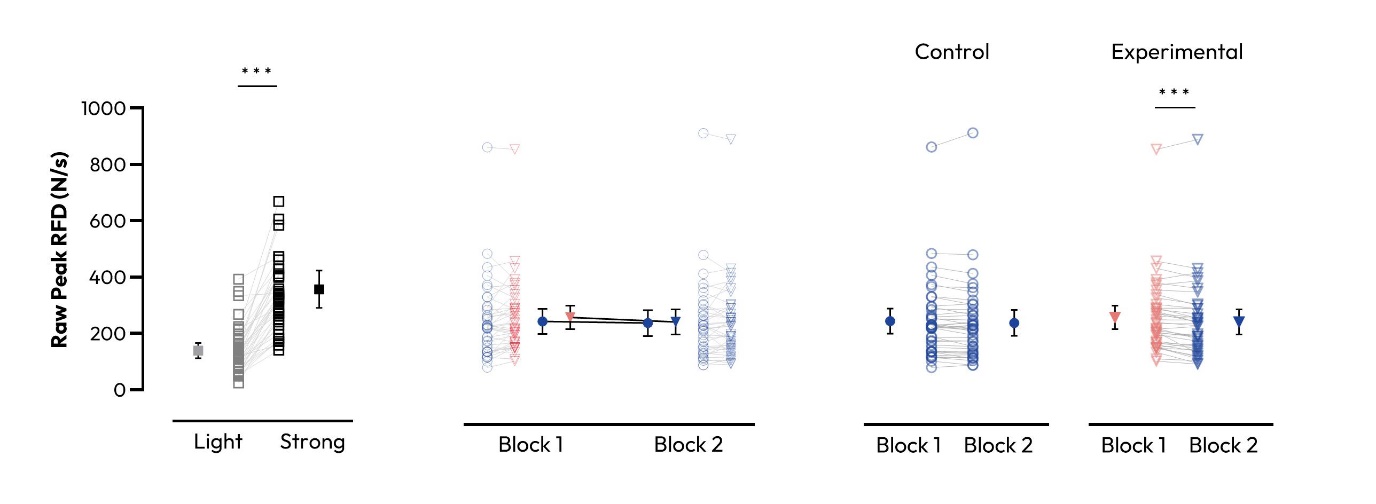


Figure S2. Changes in raw peak rate of force development between 13 ‘light’ (grey squares) and 50 ‘strong’ (black squares) fixed perceived effort intensities as well as condition $\times$ block effects in the same measures between the control (circles) and experimental (triangles) at block 1 and block 2. Red triangles indicate when the painful stimulation was applied in the experimental condition whereas blue triangles and circles indicate when the warm control stimulation was applied in the control and experimental conditions. Grey lines represent comparisons of the same participant’s data across different intensities and condition $\times$ block. Colour filled icons represent group mean data with error bars depicting 95% confidence intervals. One symbol (🞷) represents $p<.05$, two symbols represent $p<.01$, and three symbols represent $p<.001$. Denotation of ns represents non-significant findings.


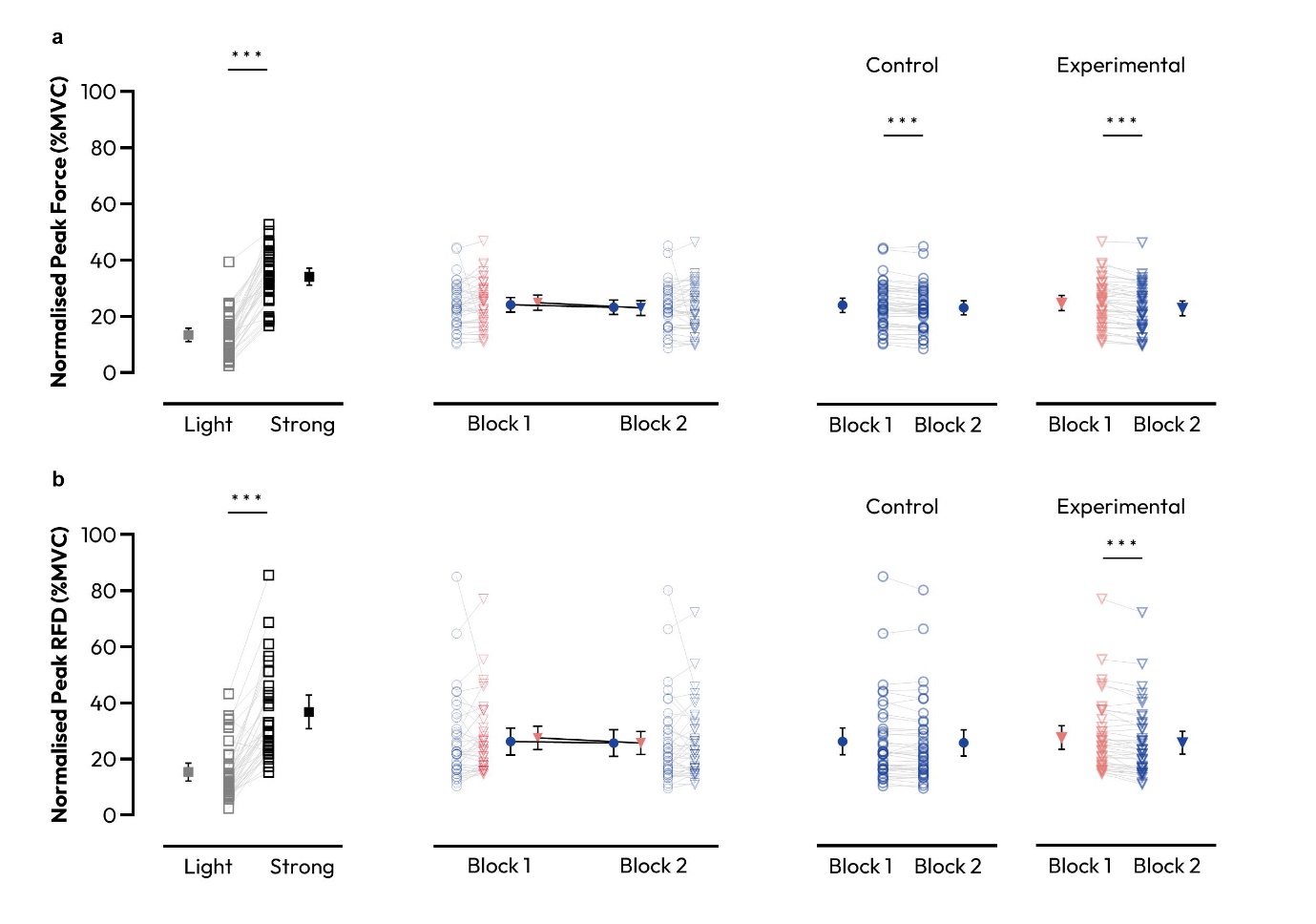
Figure S3. Changes in normalised peak force (a) and peak rate of force development (b) between 13 ‘light’ (grey squares) and 50 ‘strong’ (black squares) fixed perceived effort intensities as well as condition $\times$ block effects in the same measures between the control (circles) and experimental (triangles) at block 1 and block 2. Red triangles indicate when the painful stimulation was applied in the experimental condition whereas blue triangles and circles indicate when the warm control stimulation was applied in the control and experimental conditions. Grey lines represent comparisons of the same participant’s data across different intensities and condition $\times$ block. Colour filled icons represent group mean data with error bars depicting 95% confidence intervals. One symbol (🞷) represents $p<.05$, two symbols represent $p<.01$, and three symbols represent $p<.001$. Denotation of ns represents non-significant findings.


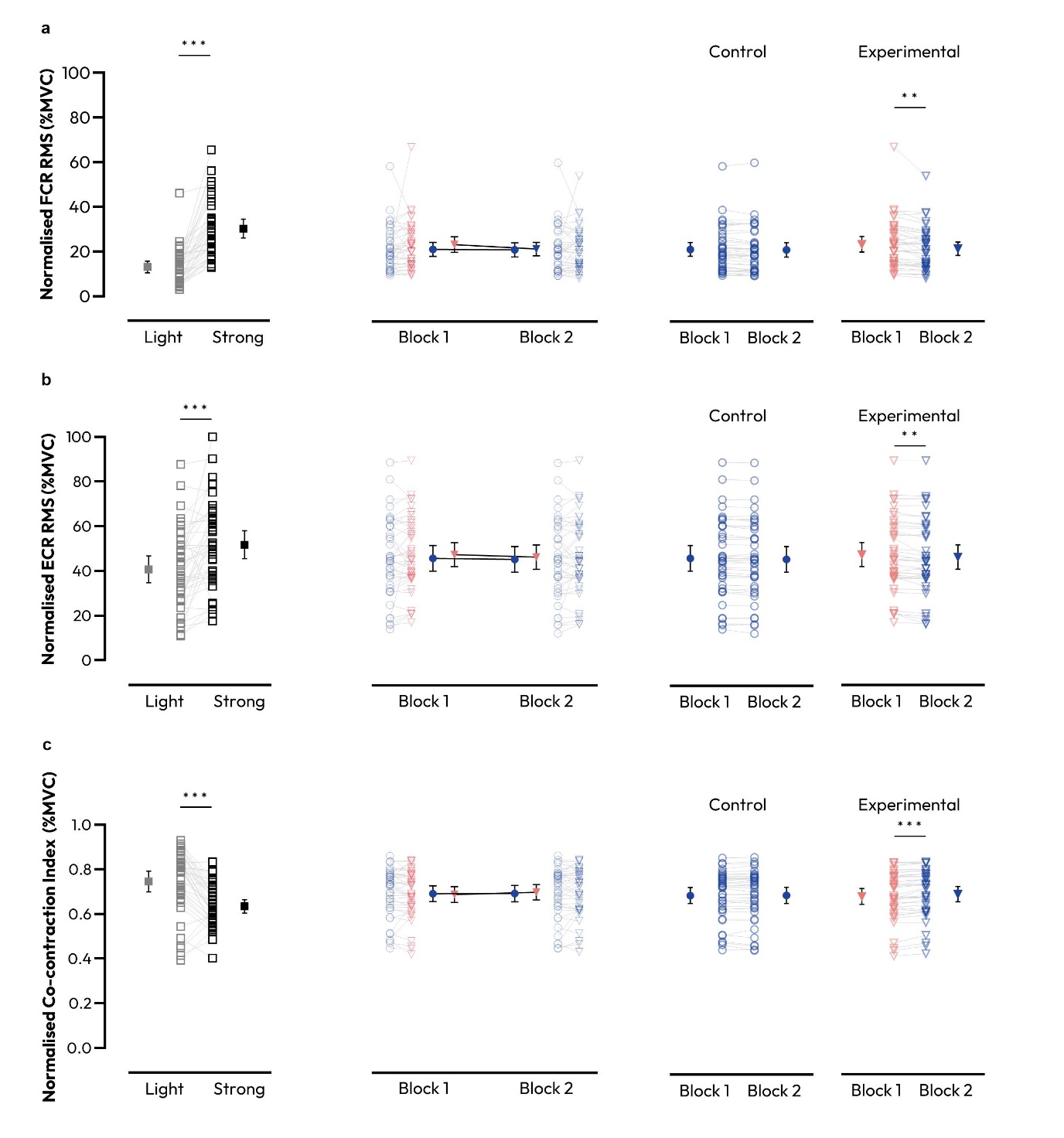
Figure S4. Changes in normalised agonist (a) antagonist (b) and co-contraction index (c) between 13 ‘light’ (grey squares) and 50 ‘strong’ (black squares) fixed perceived effort intensities as well as condition $\times$ block effects in the same measures between the control (circles) and experimental (triangles) at block 1 and block 2. Red triangles indicate when the painful stimulation was applied in the experimental condition whereas blue triangles and circles indicate when the warm control stimulation was applied in the control and experimental conditions. Grey lines represent comparisons of the same participant’s data across different intensities and condition $\times$ block. Colour filled icons represent group mean data with error bars depicting 95% confidence intervals. One symbol (🞷) represents $p<.05$, two symbols represent $p<.01$, and three symbols represent $p<.001$. Denotation of ns represents non-significant findings.


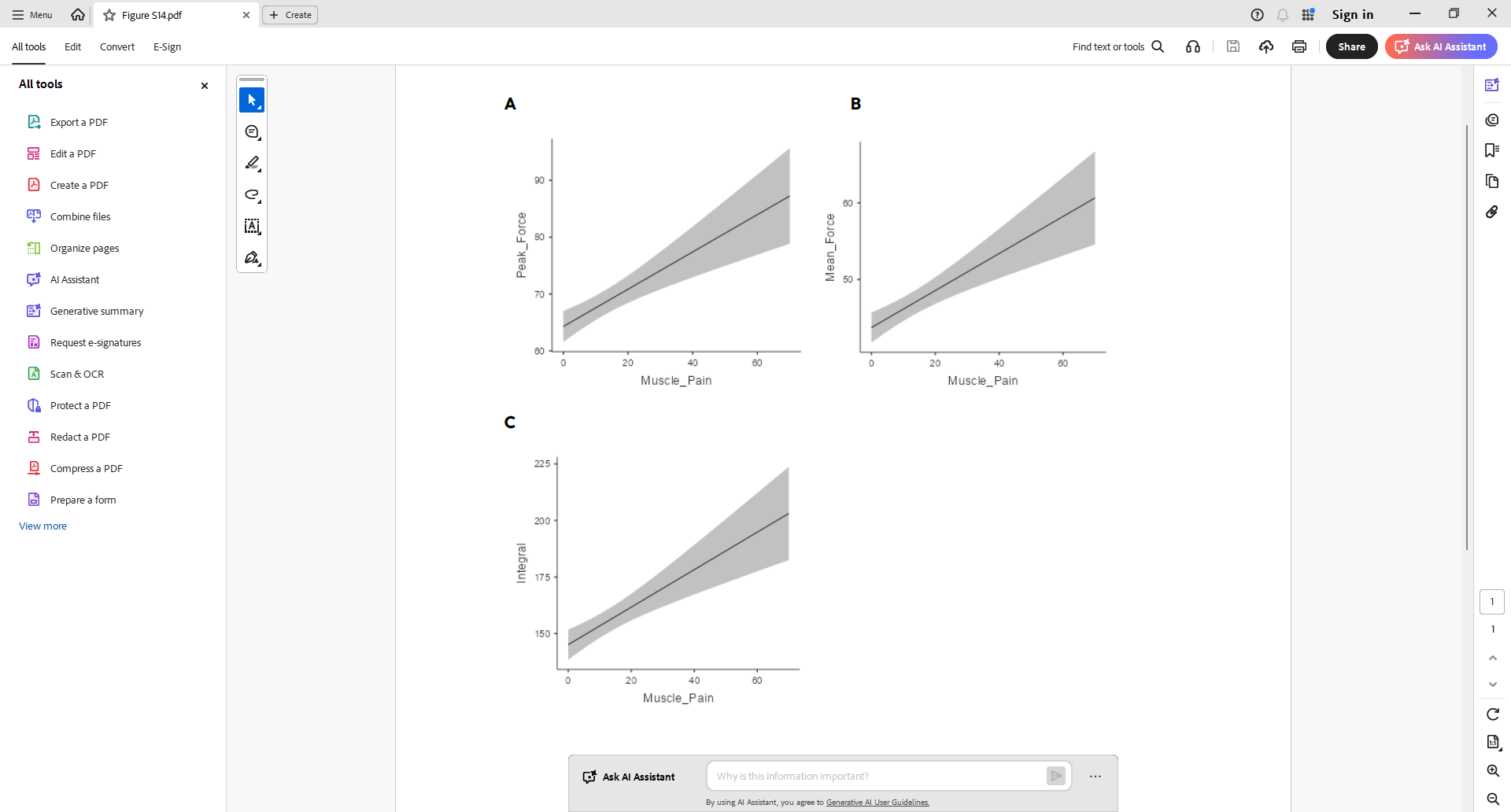


Figure S5. Changes in raw peak force (a) mean force (b), and force-time integral (c) according to changes in muscle pain ratings. Analysed data are averaged across all intensities, conditions, blocks and repetitions. The regression model included intensity and sex to control for their interactions. The thick black line denotes the main effect. The grey shaded area depicts the 95% confidence intervals. Peak force and mean force are represented in Newtons (N) whereas force-time integral is represented in Newtons over time (N•s^-1^). Muscle pain ratings are in arbitrary rating units (n).


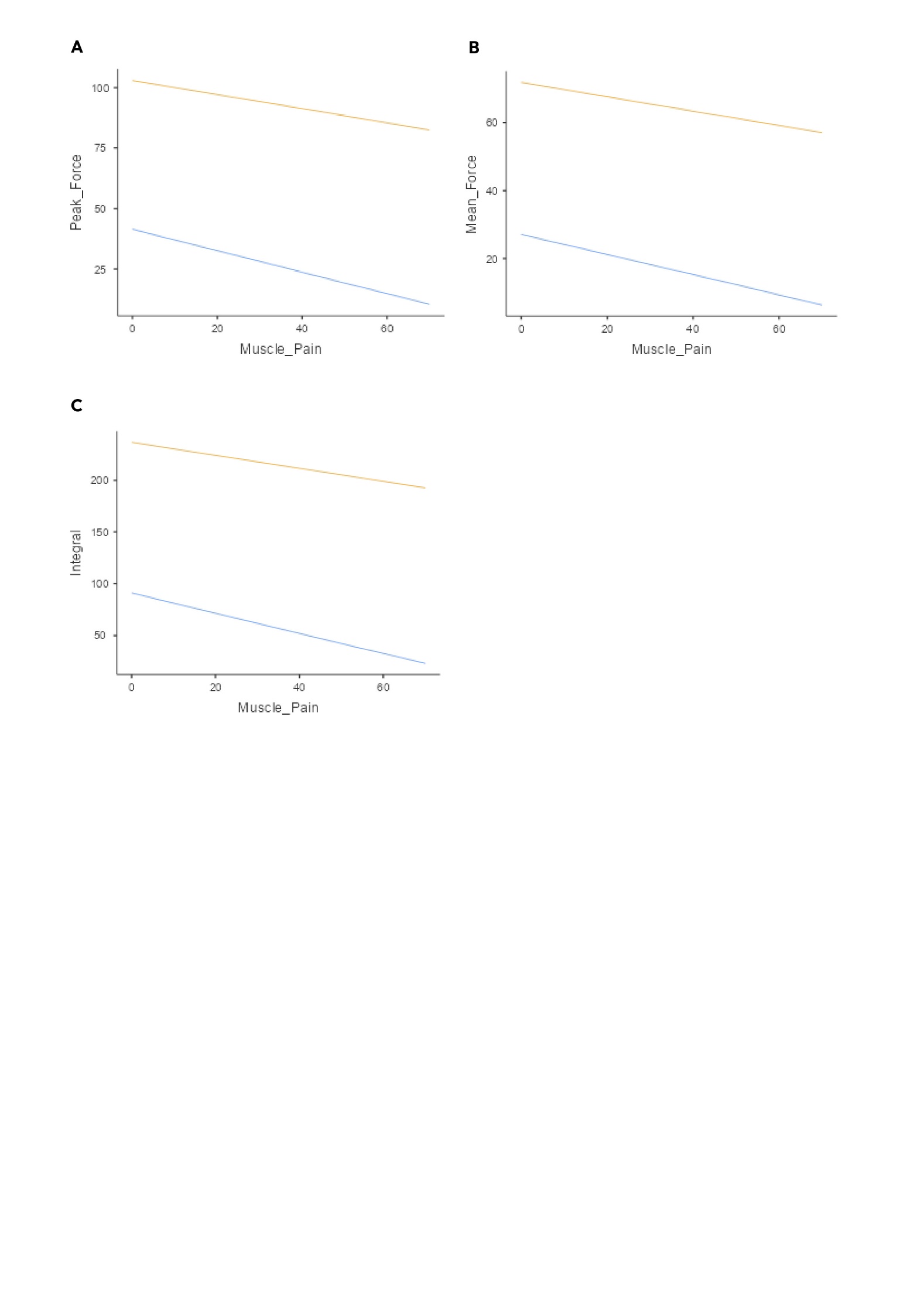


Figure S6. Changes in raw peak force (a) mean force (b), and force-time integral (c) according to changes in muscle pain ratings at light (blue line) and strong (yellow line) fixed perceived effort intensities. Analysed data are averaged across all conditions, blocks and repetitions. The regression model included intensity and sex to control for their interactions. Peak force and mean force are represented in Newtons (N) whereas force-time integral is represented in Newtons over time (N.s^-1^). Muscle pain ratings are in arbitrary rating units (n).


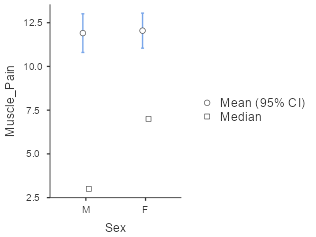


Figure S7. Differences in mean (circles) and median (squares) muscle pain ratings between male (M) and female (F) participants. Analysed data are averaged across all intensities, conditions, blocks and repetitions. Mann Whitney U tests identified a significant effect of participant sex for muscle pain ratings $(p=.001)$. Error bars represent 95% confidence intervals.


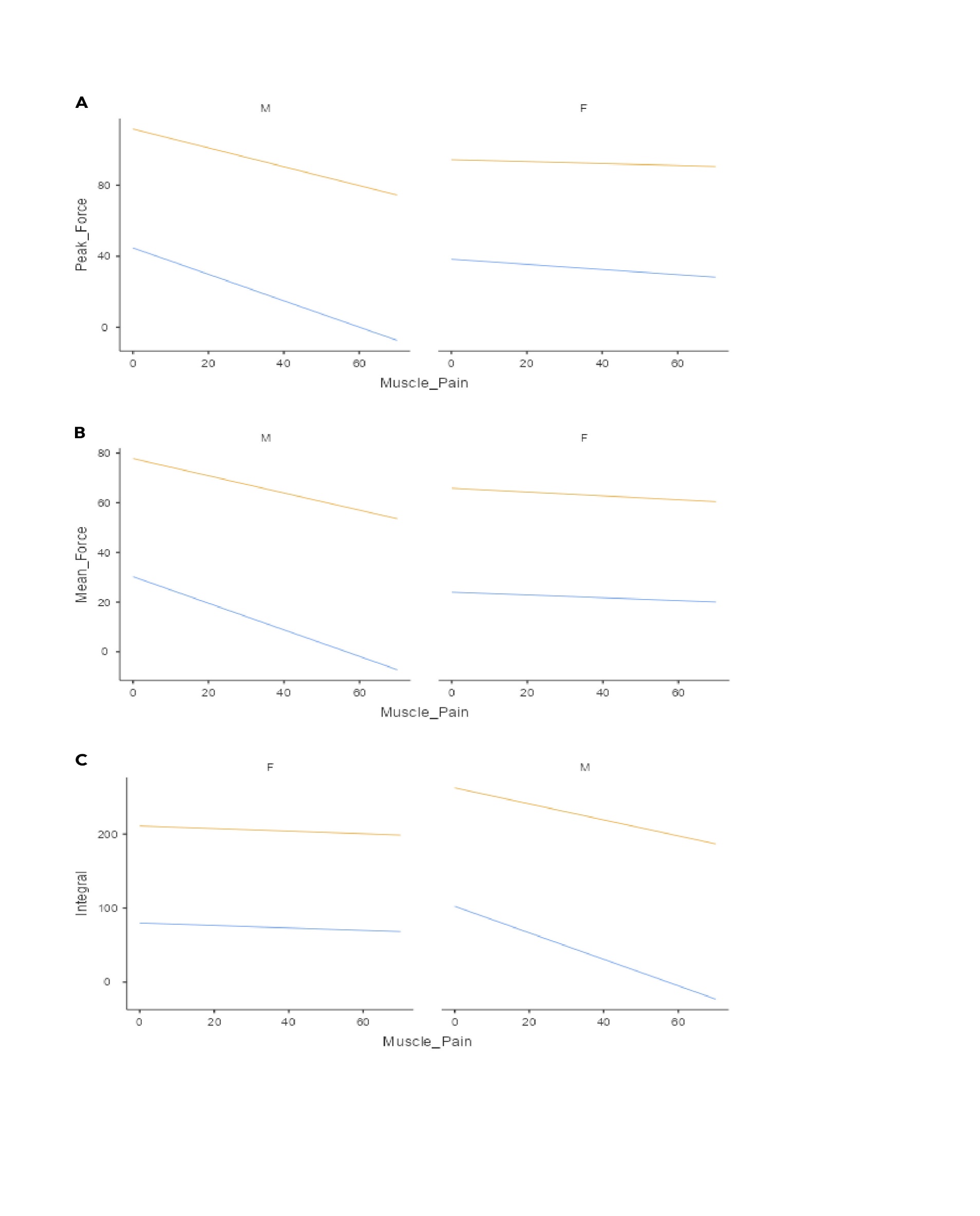


Figure S8. Changes in raw peak force (a) mean force (b), and force-time integral (c) according to changes in muscle pain ratings between participant sexes (male [M] and female [F]) at light (blue line) and strong (yellow line) fixed perceived effort intensities. Analysed data are averaged across all conditions, blocks and repetitions. Peak force and mean force are represented in Newtons (N) whereas force-time integral is represented in Newtons over time (N.s^-1^). Muscle pain ratings are in arbitrary rating units (n).


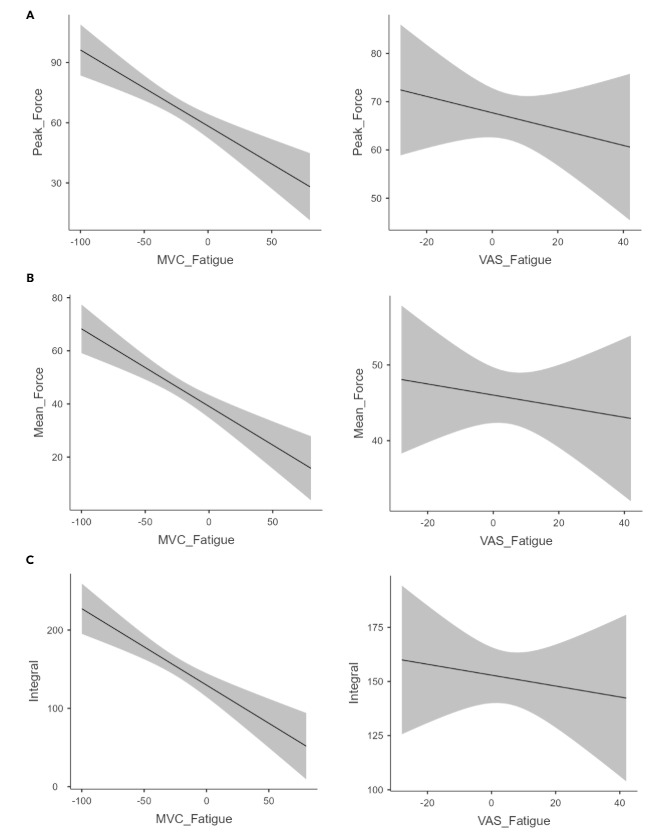


Figure S9. Changes in raw peak force (a) mean force (b), and force-time integral (c) according to changes in objective (changes in MVC peak force pre-post condition [MVC_Fatigue]) and subjective (changes in visual analogue scale ratings of fatigue pre-post condition [VAS_Fatigue]) measures of fatigue. Analysed data are averaged across both conditions. The grey shaded area depicts the 95% confidence intervals. Peak force and mean force are represented in Newtons (N) whereas force-time integral is represented in Newtons over time (N.s^-1^). MVC_Fatigue is represented in Newtons are in arbitrary rating units (n). VAS_Fatigue is represented in arbitrary units (n).


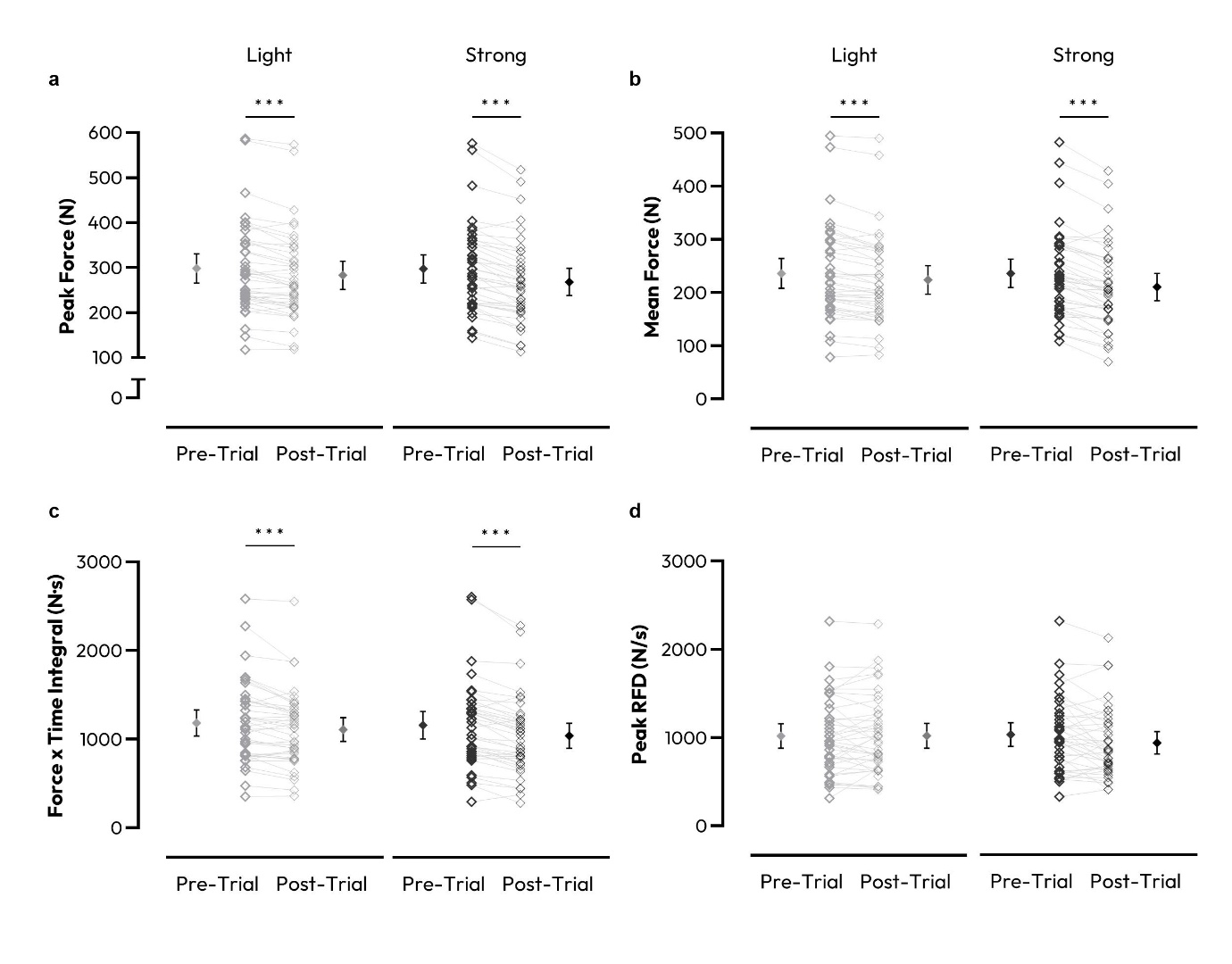
Figure S10. Pre- to post-trial comparisons of raw peak force (a), mean force (b), force-time integral (c), and peak rate of force development (d). Specific comparisons are depicted between light and strong fixed perceived effort intensities. Each data point represents the average of both pre-trial (darker shaded rhombi) and post-trial (lighter shaded rhombi) across both conditions. Grey lines represent comparisons of the same participant’s data across different time-points. Colour filled icons represent group mean data with error bars depicting 95% confidence intervals. One symbol (🞷) represents $p<.05$, two symbols represent $p<.01$, and three symbols represent $p<.001$. Denotation of ns represents non-significant findings.


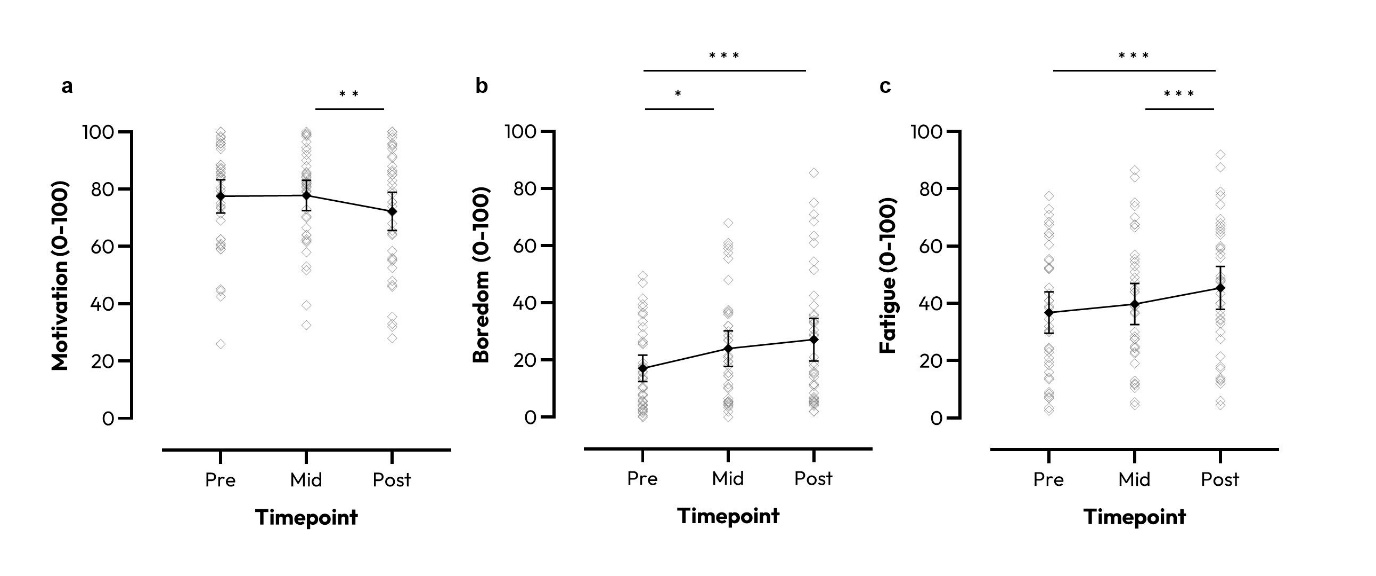
Figure S11. Pre-, mid-, and post-trial comparisons of perceived motivation (a), boredom (b), and fatigue (c). Each data point represents the average of data for each time point across both intensities and conditions. Grey icons represent individual participant data. Black icons represent group mean data with the black line linking group mean changes over each time-point. lines represent comparisons of the same participant’s data across different timepoints with error bars depicting 95% confidence intervals. One symbol (🞷) represents $p<.05$, two symbols represent $p<.01$, and three symbols represent $p<.001$. Denotation of ns represents non-significant findings.

There were no condition $\times$ block interactions for the number of false starts to the contract $(p=.511)$ and relax $(p=.903)$ prompt.


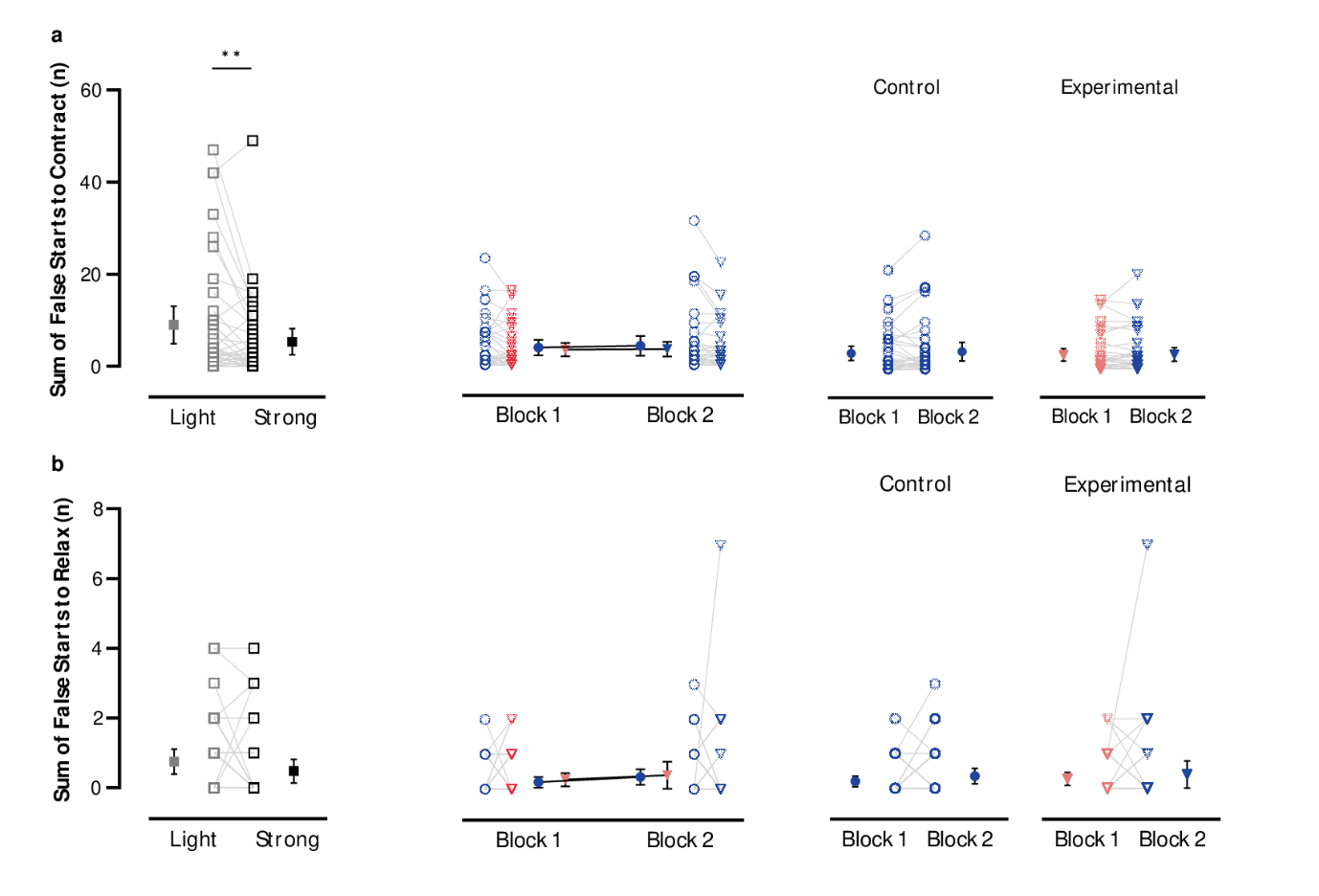
Figure S12. Changes in the sum of false starts to the contract (a) and relax (b) false prompt between 13 ‘light’ (grey squares) and 50 ‘strong’ (black squares) fixed perceived effort intensities as well as condition $\times$ block effects in the same measures between the control (circles) and experimental (triangles) at block 1 and block 2. Red triangles indicate when the painful stimulation was applied in the experimental condition whereas blue triangles and circles indicate when the warm control stimulation was applied in the control and experimental conditions. Grey lines represent comparisons of the same participant’s data across different intensities and condition $\times$ block. Colour filled icons represent group mean data with error bars depicting 95% confidence intervals. One symbol (🞷) represents $p<.05$, two symbols represent $p<.01$, and three symbols represent $p<.001$. Denotation of ns represents non-significant findings.

A large condition $\times$ block effect was observed for reaction time to the contract prompt $\left( F_{1,34}=4.944,p=.033,\eta_{p}^{2}=.127[.000-.333] \right)$(Figure 5a). Pairwise comparisons between experimental and control block 1 showed no differences in the reaction time to the contract prompt $(t_{39}=-1.359,p=1.000,d=-0.124)$. In the experimental condition, reaction time to the contract prompt was higher in block 1 than block 2 but only approached significance $(t_{39}=-3.301,p=.054,d=0.177)$. A large condition $\times$ block effect was observed for the reaction time to the relax prompt. Pairwise comparisons between experimental and control block 1 showed no differences in the reaction time to the relax prompt $(t_{39}=-1.329,p=.192,d=0.069)$. In the experimental condition, reaction time to the relax prompt was higher in block 1 than block 2 $\left( t_{39}=5.493,p<.001, d=0.194[0.078-0.310] \right)$.

Contraction duration showed a large condition $\times$ block effect. Contraction duration was longer in experimental block 1 than control block 1 $\left( t_{39}=-2.245,p=.031, d=0.121[0.034-0.275] \right)$. Contraction duration was also higher in experimental block 1 than experimental block 2 $\left( t_{39}=4.569,p<.001, d=0.282[0.089-0.476] \right)$.


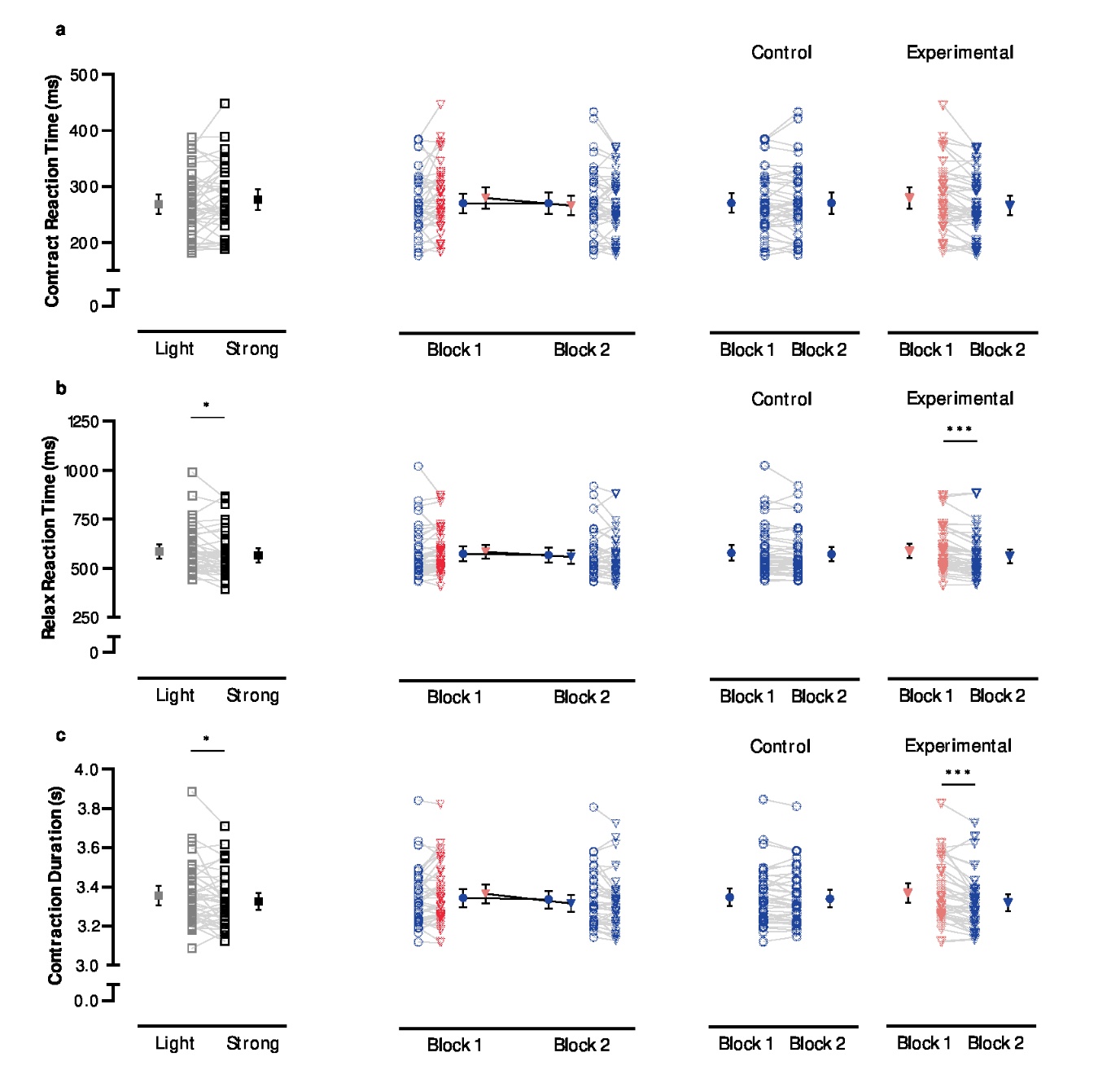


Figure S13. Changes in mean contract reaction time (a), mean relax reaction time (b), and contraction duration (c) between light (grey squares) and strong (black squares) fixed perceived effort intensities as well as condition × block effects in the same measures between the control (circles) and experimental (triangles) at block 1 and block 2. Red triangles indicate when the painful stimulation was applied in the experimental condition whereas blue triangles and circles indicate when the warm control stimulation was applied in the control and experimental conditions. Grey lines represent comparisons of the same participant’s data across different intensities and condition × block. Colour filled icons represent group mean data with error bars depicting 95% confidence intervals. One symbol () represents p<.05, two symbols represent p<.01, and three symbols represent p<.001. Denotation of ns represents non-significant findings

No condition $\times$ block interaction effects for heart rate $\left( F_{1,36}=10.077,p=.260,\eta_{p}^{2}=.035 \right)$ or breathing frequency $\left( F_{1,38}=0.005,p=.317,\eta_{p}^{2}=.026 \right)$ were observed.


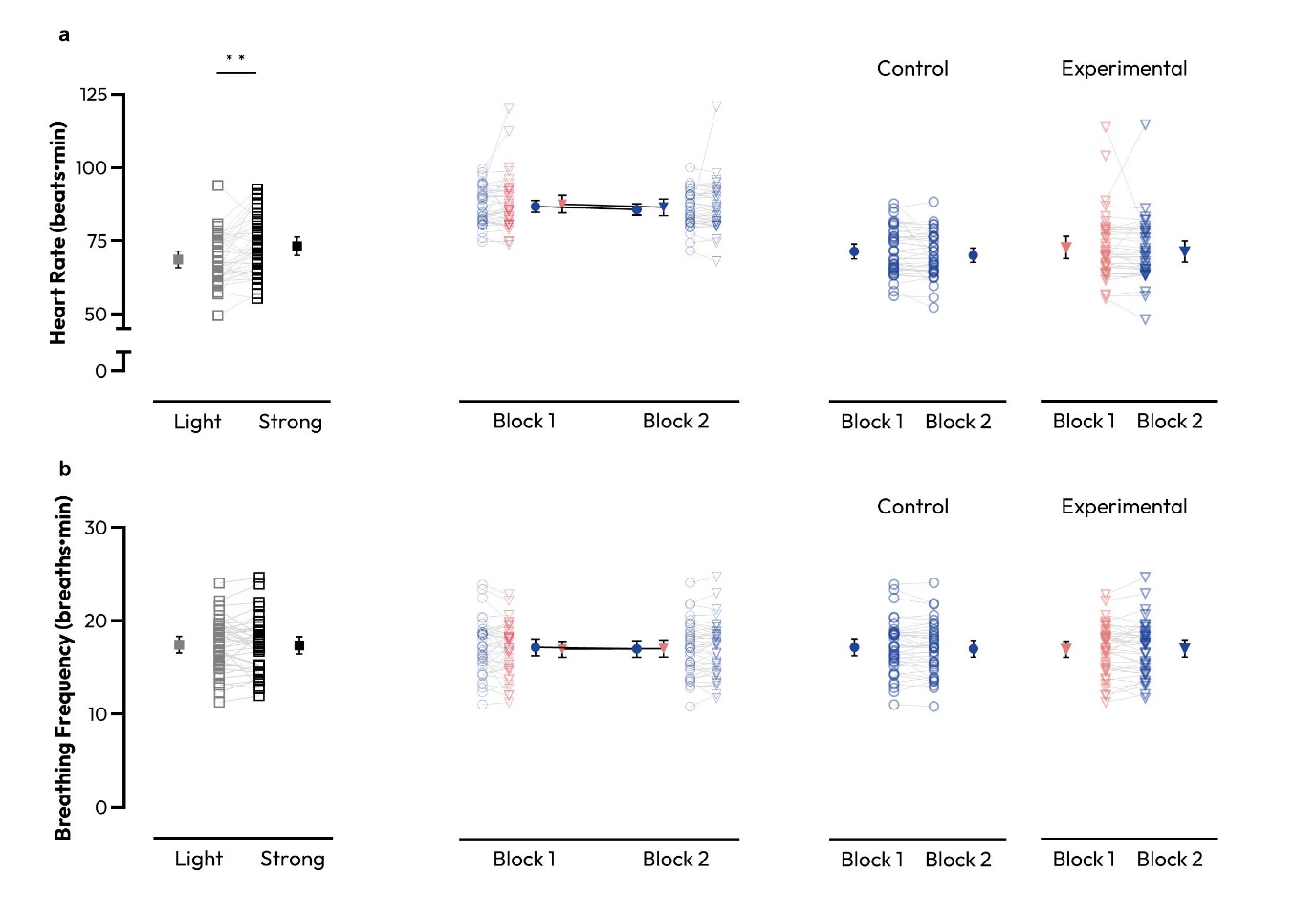
Figure S14. Changes in mean heart rate (a) and breathing frequency (b) between 13 ‘light’ (grey squares) and 50 ‘strong’ (black squares) fixed perceived effort intensities as well as condition $\times$ block effects in the same measures between the control (circles) and experimental (triangles) at block 1 and block 2. Red triangles indicate when the painful stimulation was applied in the experimental condition whereas blue triangles and circles indicate when the warm control stimulation was applied in the control and experimental conditions. Grey lines represent comparisons of the same participant’s data across different intensities and condition $\times$ block. Colour filled icons represent group mean data with error bars depicting 95% confidence intervals. One symbol (🞷) represents $p<.05$, two symbols represent $p<.01$, and three symbols represent $p<.001$. Denotation of ns represents non-significant findings.

Repetition main effects were observed for muscle pain with a large effect size. Muscle pain at repetition 1 was lower than all other repetitions $(p's<.001,d's=0.111-0.336)$. Muscle pain was higher in repetition 5 than all other repetitions $\left( p's<.001,d's=0.088-0.225 \right)$ suggesting muscle pain increased with time-on-task throughout each repetition.


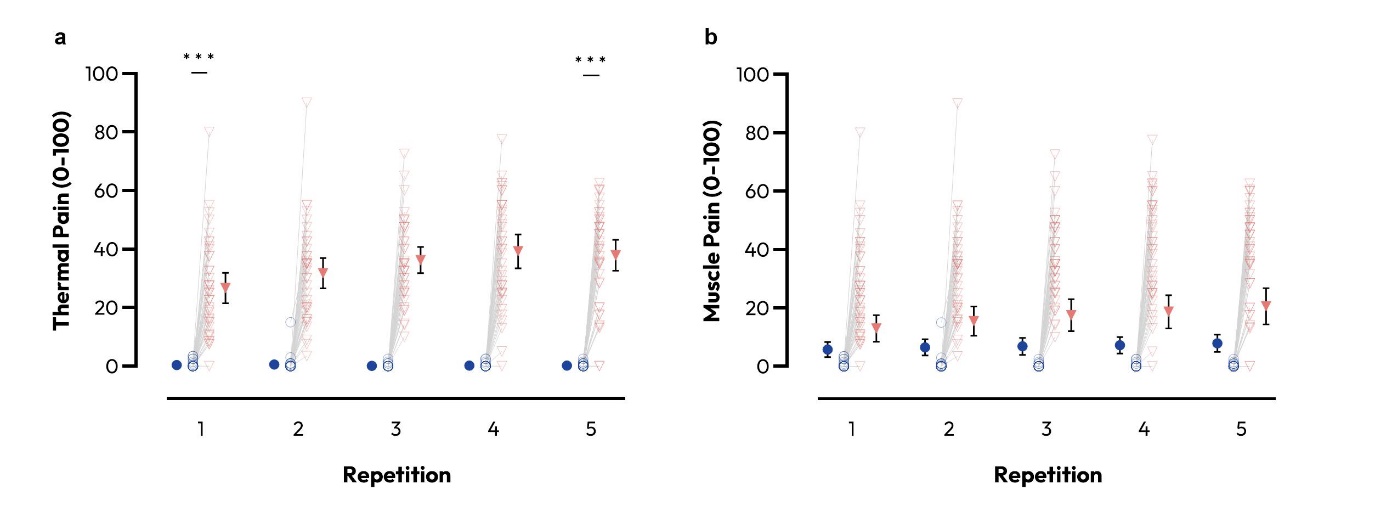
Figure S15. Changes in thermal pain (a) and muscle pain (b) ratings indexing pairwise comparisons of repetition 1 and 5 condition $\times$ block $\times$ interaction effects. Specific comparisons are depicted between control (circles) and experimental (triangles) at block 1 across each repetition. Each data point represents the average of both light and strong intensities at block 1. Red triangles indicate when the painful stimulation was applied in the experimental condition whereas blue circles indicate when the warm control stimulation was applied in the control condition. Grey lines represent comparisons of the same participant’s data across different condition $\times$ block. Colour filled icons represent group mean data with error bars depicting 95% confidence intervals. One symbol (🞷) represents $p<.05$, two symbols represent $p<.01$, and three symbols represent $p<.001$. Denotation of ns represents non-significant findings.

Repetition main effects were observed for all force parameters with large effect sizes. All force measures were higher at repetition 1 compared to all other repetitions $\left( p's<.001, d's=0.157-0.424 \right)$. All force measures at repetition 2 were higher than repetition 5 $\left( p^{'}s<.017, d's=0.092-0.130 \right)$. There were no differences between repetitions 3 $(p's>.152,d's<0.033)$ and 4 $(p's>.087,d's<0.033)$ to repetition 5 indicating a plateau in force measures occurred at repetition 3.


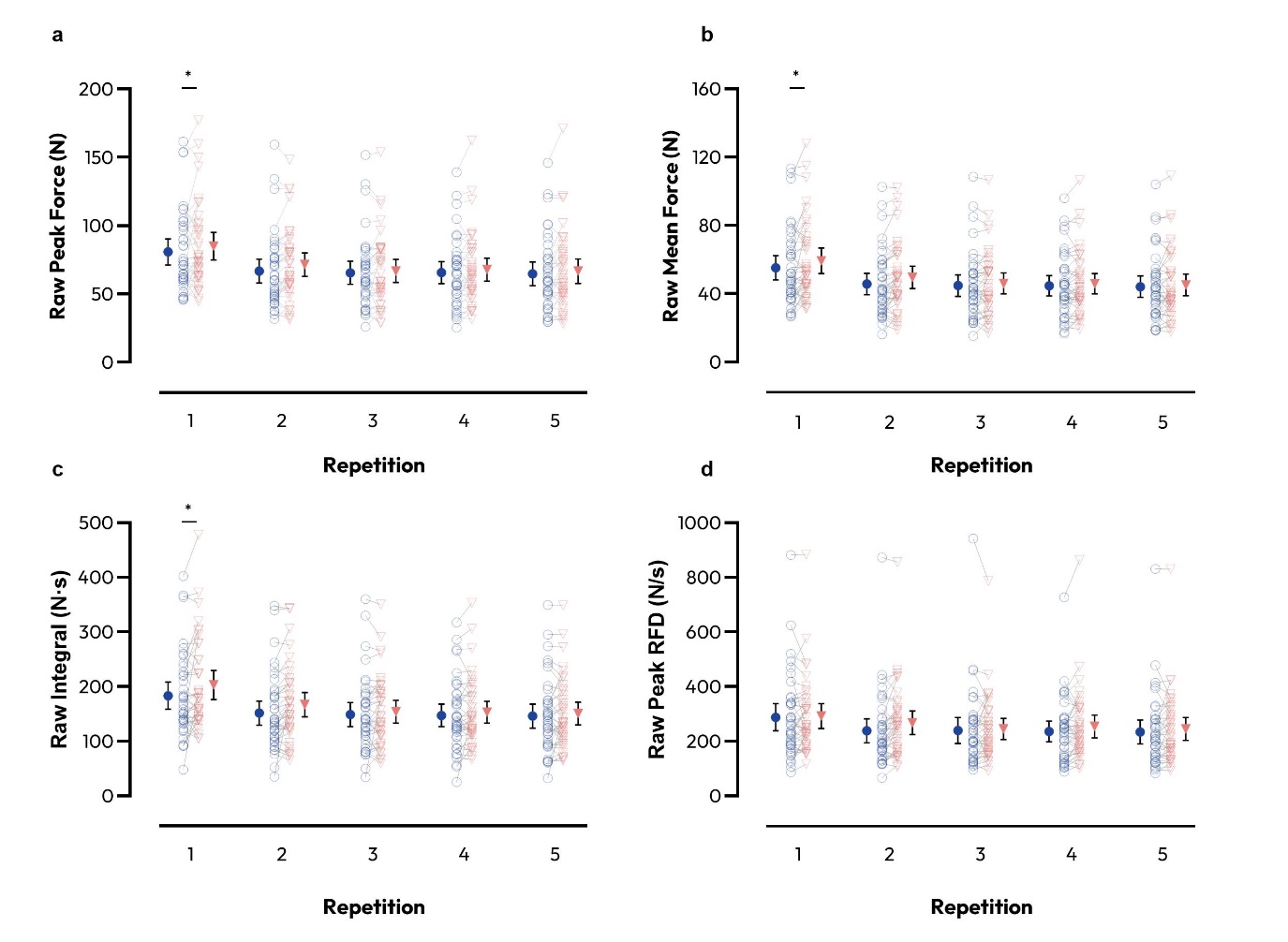
Figure S16. Changes in raw peak force (a), mean force (b), force-time integral (c), and peak rate of force development (d) indexing pairwise comparisons of repetition 1 and 5 condition $\times$ block $\times$ interaction effects. Specific comparisons are depicted between control (circles) and experimental (triangles) at block 1 across each repetition. Each data point represents the average of both light and strong intensities at block 1. Red triangles indicate when the painful stimulation was applied in the experimental condition whereas blue circles indicate when the warm control stimulation was applied in the control condition. Grey lines represent comparisons of the same participant’s data across different condition $\times$ block. Colour filled icons represent group mean data with error bars depicting 95% confidence intervals. One symbol (🞷) represents $p<.05$, two symbols represent $p<.01$, and three symbols represent $p<.001$. Denotation of ns represents non-significant findings.


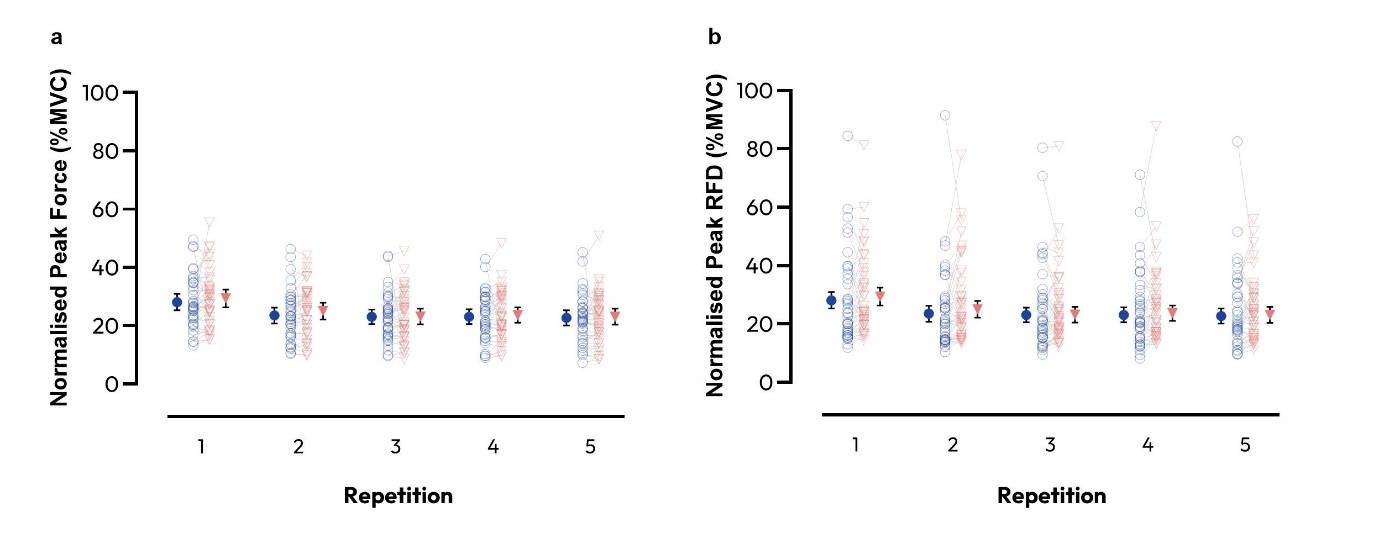
Figure S17. Changes in normalised peak force (a) and peak rate of force development (b) indexing pairwise comparisons of repetition 1 and 5 condition $\times$ block $\times$ interaction effects. Specific comparisons are depicted between control (circles) and experimental (triangles) at block 1 across each repetition. Each data point represents the average of both light and strong intensities at block 1. Red triangles indicate when the painful stimulation was applied in the experimental condition whereas blue circles indicate when the warm control stimulation was applied in the control condition. Grey lines represent comparisons of the same participant’s data across different condition $\times$ block. Colour filled icons represent group mean data with error bars depicting 95% confidence intervals. One symbol (🞷) represents $p<.05$, two symbols represent $p<.01$, and three symbols represent $p<.001$. Denotation of ns represents non-significant findings.

Repetition main effects were observed for all EMG measures with large effect sizes. The raw and normalised EMG measures of both muscles were higher during repetition 1 versus all other repetitions $\left( p's<.017, d's=0.039-0.206 \right)$. There were no differences between raw and normalised EMG measures at repetition 5 to all other repetitions suggests EMG activity plateaued at repetition 2 $(p's>.058,d's<0.079)$. There were no differences in the raw co-contraction index measure at between any repetitions $(p's>.054,d's<0.082)$.


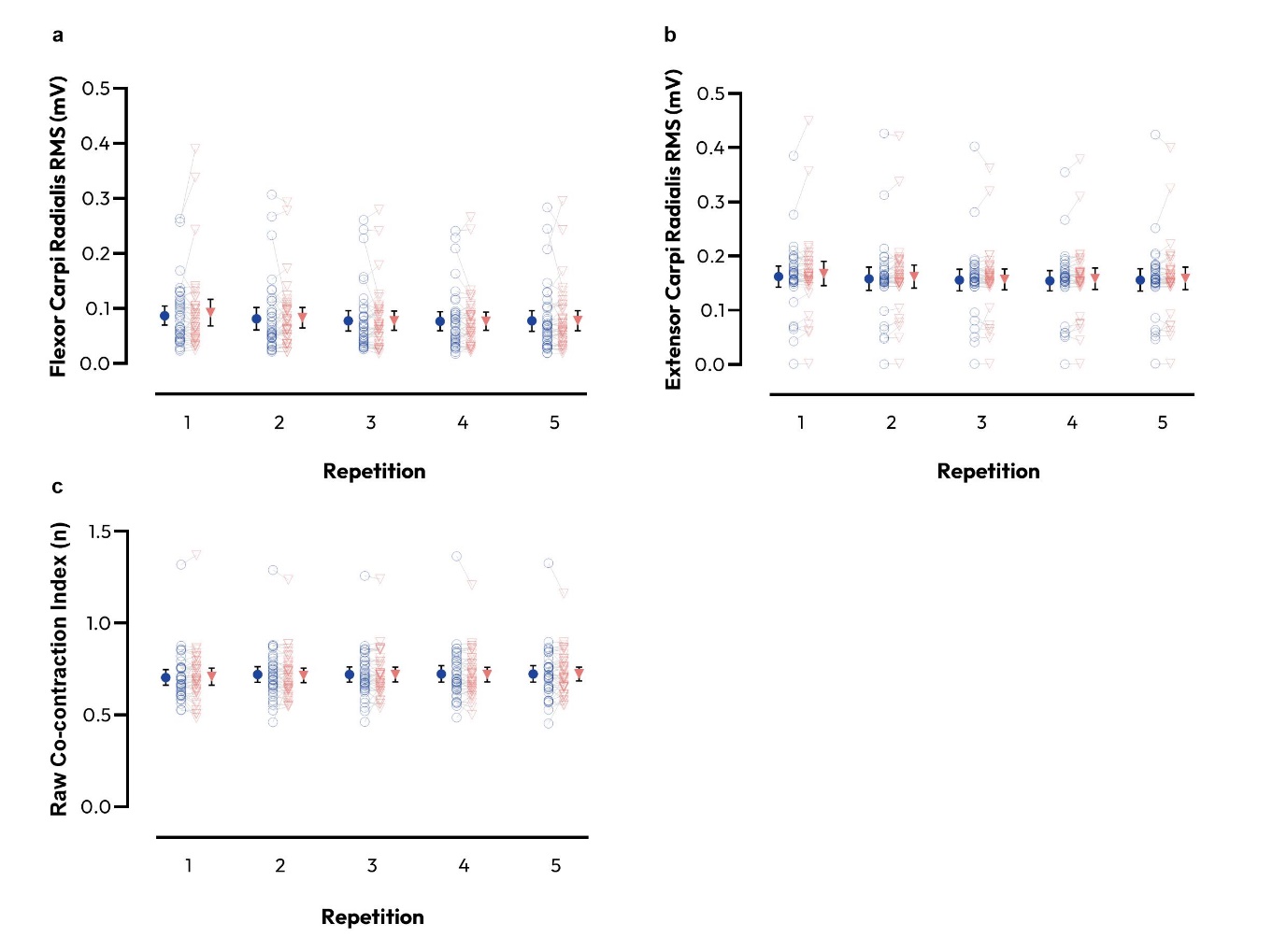
Figure S18. Changes in raw agonist (a), antagonist (b), and co-contraction index (c) indexing pairwise comparisons of repetition 1 and 5 condition $\times$ block $\times$ interaction effects. Specific comparisons are depicted between control (circles) and experimental (triangles) at block 1 across each repetition. Each data point represents the average of both light and strong intensities at block 1. Red triangles indicate when the painful stimulation was applied in the experimental condition whereas blue circles indicate when the warm control stimulation was applied in the control condition. Grey lines represent comparisons of the same participant’s data across different condition $\times$ block. Colour filled icons represent group mean data with error bars depicting 95% confidence intervals. One symbol (🞷) represents $p<.05$, two symbols represent $p<.01$, and three symbols represent $p<.001$. Denotation of ns represents non-significant findings.

Pairwise comparisons show that normalised co-contraction index was lower at repetition 1 versus repetitions 3, 4, and 5 $\left( p^{'}s<.022, d's=0.085-0.114 \right)$ implying that participants’ had less efficient muscle activation as time-on-task increased, likely due to fatigue-related effects.


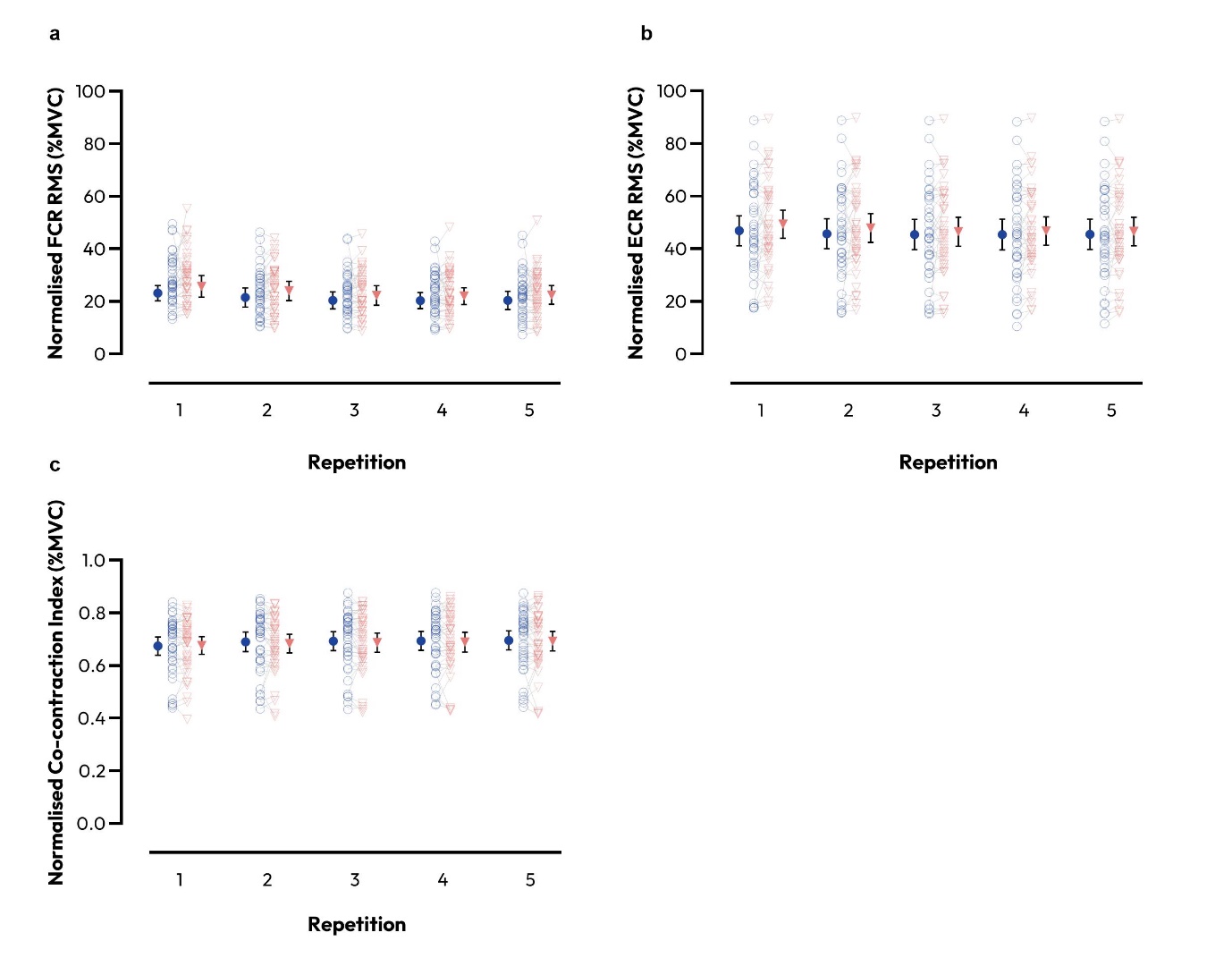
Figure S19. Changes in normalised agonist (a), antagonist (b), and co-contraction index (c) indexing pairwise comparisons of repetition 1 and 5 condition $\times$ block $\times$ interaction effects. Specific comparisons are depicted between control (circles) and experimental (triangles) at block 1 across each repetition. Each data point represents the average of both light and strong intensities at block 1. Red triangles indicate when the painful stimulation was applied in the experimental condition whereas blue circles indicate when the warm control stimulation was applied in the control condition. Grey lines represent comparisons of the same participant’s data across different condition $\times$ block. Colour filled icons represent group mean data with error bars depicting 95% confidence intervals. One symbol (🞷) represents $p<.05$, two symbols represent $p<.01$, and three symbols represent $p<.001$. Denotation of ns represents non-significant findings.

A main repetition effect was observed for false starts to the contract prompt. However, pairwise comparisons showed no notable differences between specific repetitions $(p^{'}s<.310)$. No main effects of repetition were observed for false starts to the relax prompts. There was no main effect of repetition on reaction time to the contract prompt. There was a main effect of repetition on reaction time to the relax prompts with large effects. Reaction time to the relax prompt was slower in repetition 1 than all other repetitions $(p^{'}s<.001,d's=0.176-0.246)$. There were no differences in reaction time to the relax prompt between any other repetitions $(p's>.180,d's<0.060)$. There was no main effect of repetition on contraction duration.


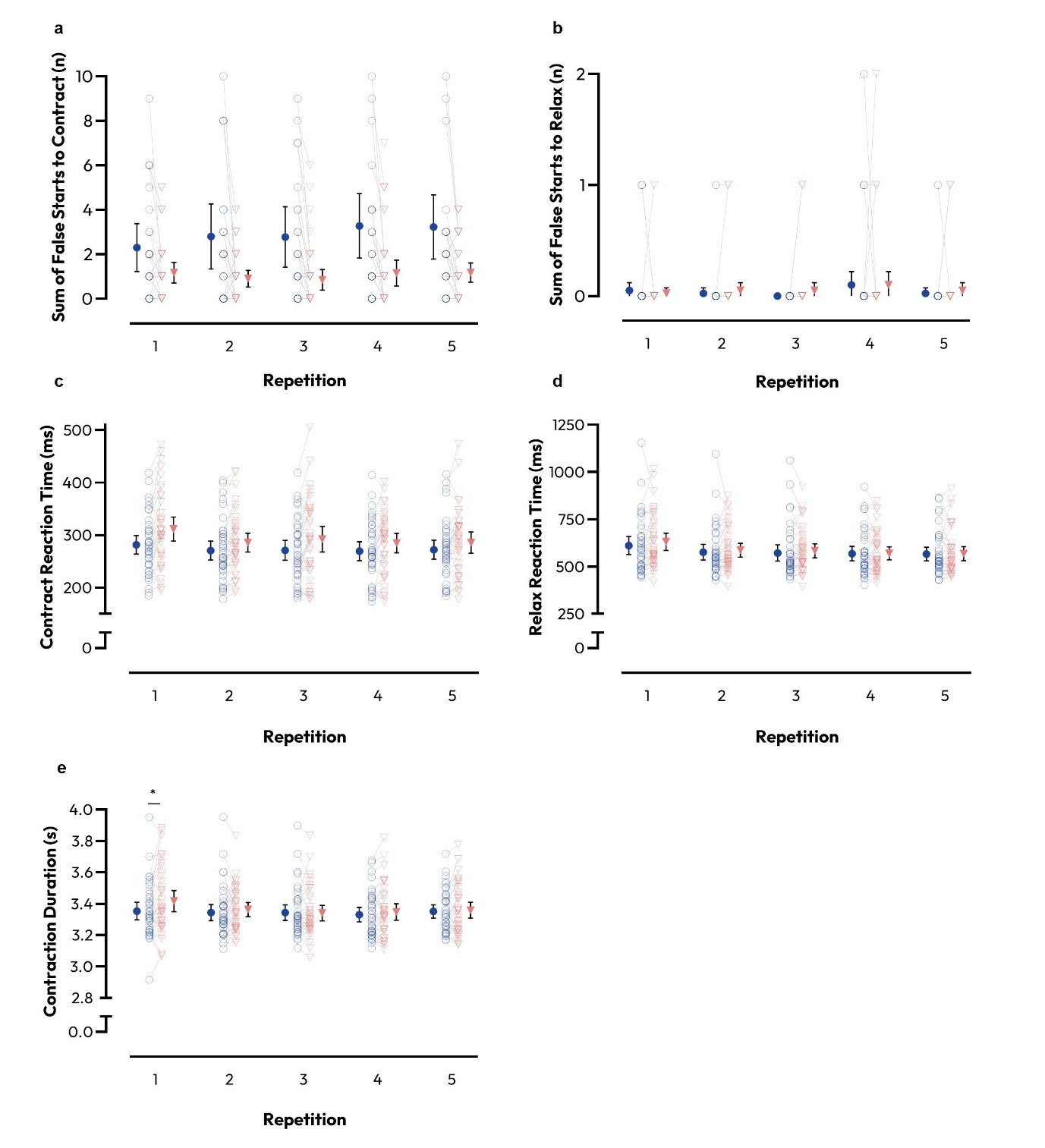
Figure S20. Changes in sum of false starts to the contract (a) and relax prompts (b), contract (c) and relax (d) response times, and contraction duration (e) indexing pairwise comparisons of repetition 1 and 5 condition $\times$ block $\times$ interaction effects. Specific comparisons are depicted between control (circles) and experimental (triangles) at block 1 across each repetition. Each data point represents the average of both light and strong intensities at block 1. Red triangles indicate when the painful stimulation was applied in the experimental condition whereas blue circles indicate when the warm control stimulation was applied in the control condition. Grey lines represent comparisons of the same participant’s data across different condition $\times$ block. Colour filled icons represent group mean data with error bars depicting 95% confidence intervals. One symbol (🞷) represents $p<.05$, two symbols represent $p<.01$, and three symbols represent $p<.001$. Denotation of ns represents non-significant findings.

Heart rate did not show a repetition main effect. Breathing frequency did show a repetition main effect with a moderate effect size. Pairwise comparisons showed that breathing frequency was higher in repetitions 3 $\left( t_{39}=2.963, p=.031, d=0.131\left[ 0.008, 0.269 \right] \right)$ and 5 $\left( t_{39}=3.754, p=.004, d=0.185\left[ 0.025, 0.344 \right] \right)$ compared to repetition 1. Breathing frequency at repetition 1 did not differ to breathing frequency at repetition 2 or 4 $(p's>.067,d's<0.138)$. Breathing frequency at repetition 5 did not differ to repetitions 2-4 $(p's>.258,d's<0.245)$.


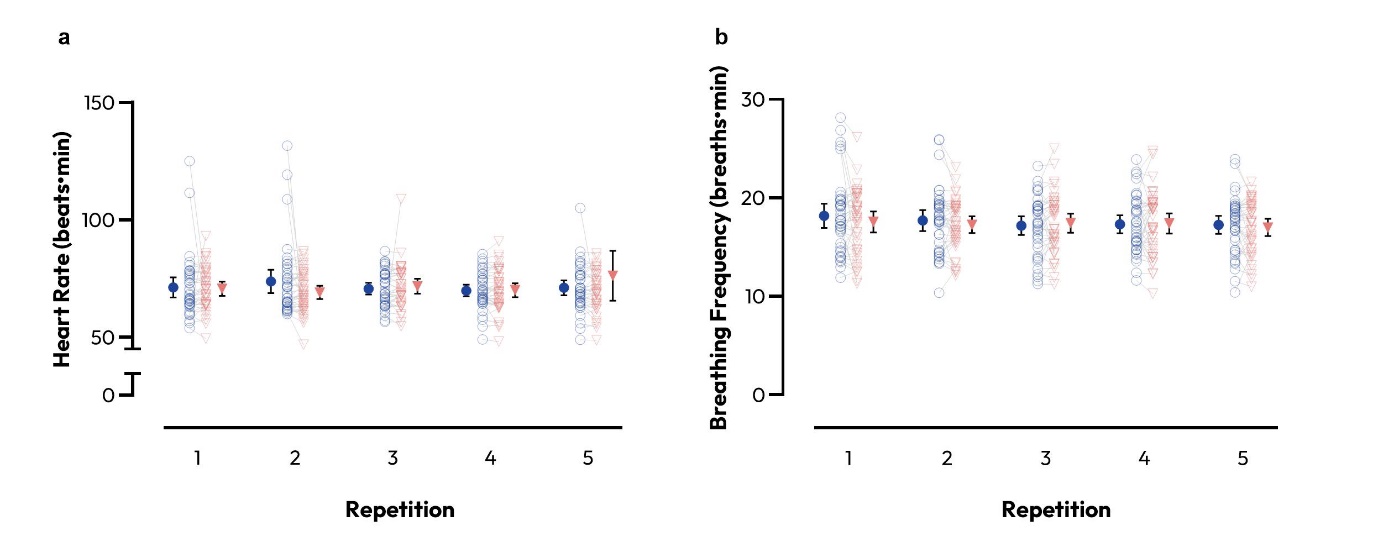
Figure S21. Changes in mean heart rate (a) and breathing frequency (b) indexing pairwise comparisons of repetition 1 and 5 condition $\times$ block $\times$ interaction effects. Specific comparisons are depicted between control (circles) and experimental (triangles) at block 1 across each repetition. Each data point represents the average of both light and strong intensities at block 1. Red triangles indicate when the painful stimulation was applied in the experimental condition whereas blue circles indicate when the warm control stimulation was applied in the control condition. Grey lines represent comparisons of the same participant’s data across different condition $\times$ block. Colour filled icons represent group mean data with error bars depicting 95% confidence intervals. One symbol (🞷) represents $p<.05$, two symbols represent $p<.01$, and three symbols represent $p<.001$. Denotation of ns represents non-significant findings.


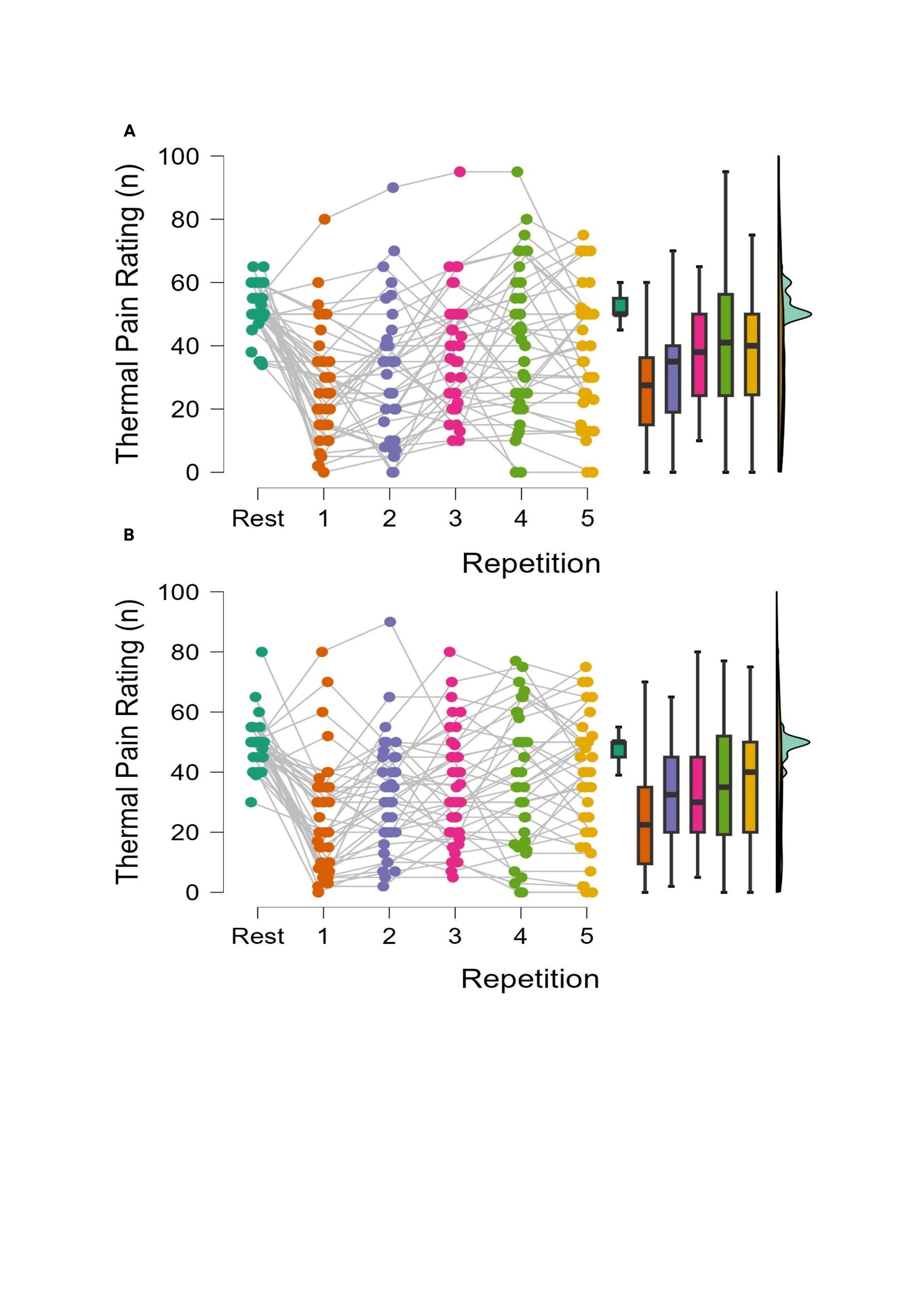


Figure S22. Changes in thermal pain ratings between time-points averaged over the light (a) and strong (b) intensties. Each data point represents the average of data for each time point across conditions and blocks. Grey lines represent comparisons of the same participant’s data across different time-points. Boxplots depict group mean data for each time-point with whiskers depicting standard deviations.

# Table S3. Repeated measures ANOVA outputs for intensity and repetition main effects and intensity x condition x block, condition x block x repetition, and intensity x condition x block x repetition interactions.

| a | | Pain Ratings | | | | | |
| --- | --- | --- | --- | --- | --- | --- | --- |
|  |  | Thermal Pain | | | Muscle Pain | | |
|  |  | $F$  $(df)$ | $p$ | $\eta_{P}^{2}$  (95%CI) | $F$  $(df)$ | $p$ | $\eta_{P}^{2}$  (95%CI) |
| Main Effects | Intensity | 0.153  (1,39) | .698 | .004  (.000-.113) | 23.734  (1,39) | <.001 | .378  (.139,.548) |
|  | Repetition | 6.569  (2.303,89.815) | .001 | .144  (.026-.264) | 23.601  (1.359,52.993) | <.001 | .377  (.170-.523) |
| Interaction Effect | Condition x Block | 183.253  (1,39) | <.001 | .825  (.706-.877) | 0.543  (1,39) | .465 | .014  (.000-.150) |
|  | Intensity x Condition x Block | 0.345  (1,39) | .560 | .009  (.000-.135) | 1.245  (1,39) | .271 | .031  (.000-.188) |
|  | Condition x Block x Repetition | 10.720  (2.928,114.208) | <.001 | .216  (.083,.325) | 0.332  (3.066,119.568) | .807 | .008  (.000-.039) |
|  | Intensity x Condition x Block x Repetition | 0.806  (2.781,108.478) | .485 | .020  (.000-.076) | 0.507  (2.720,106.061) | .660 | .013  (.000-.059) |

| b | | Force Measures | | | | | | | | | | | | | | | | | |
| --- | --- | --- | --- | --- | --- | --- | --- | --- | --- | --- | --- | --- | --- | --- | --- | --- | --- | --- | --- |
|  |  | Peak Force | | | Mean Force | | | Force-Time Integral | | | Peak RFD | | | Normalised Peak Force | | | Normalised Peak RFD | | |
|  |  | $F$  $(df)$ | $p$ | $\eta_{P}^{2}$  (95%CI) | $F$  $(df)$ | $p$ | $\eta_{P}^{2}$  (95%CI) | $F$  $(df)$ | $p$ | $\eta_{P}^{2}$  (95%CI) | $F$  $(df)$ | $p$ | $\eta_{P}^{2}$  (95%CI) | $F$  $(df)$ | $p$ | $\eta_{P}^{2}$  (95%CI) | $F$  $(df)$ | $p$ | $\eta_{P}^{2}$  (95%CI) |
| Main Effect | Intensity | 199.379  (1,39) | <.001 | .836  (.725,.885) | 197.389  (1,39) | <.001 | .835  (725,.885) | 171.167  (1,39) | <.001 | .814  (.690,.870) | 66.100  (1,39) | <.001 | .625  (.419,.739) | 263.849  (1,39) | <.001 | .871  (.782,.910) | 92.398  (1,39) | <.001 | .703  (.522,.791) |
|  | Repetition | 48.731  (2.279,88.898) | <.001 | .555  (.407,.646) | 46.082 (2.170,84.649) | <.001 | .542  (.387,637) | 44.500 (1.994,77.773) | <.001 | .533  (.370,.633) | 27.659 (1.991,77.662) | <.001 | .415  (.239,.535) | 48.402 (2.077,80.987) | <.001 | .554  (.398,.649) | 19.876 (2.059,80.308) | <.001 | .338  (.116,.465) |
| Interaction Effects | Condition x Block | 10.513  (1,39) | .002 | .212  (.031-.408) | 11.894  (1,39) | .002 | .226  (.041-.428) | 14.759  (1,39) | <.001 | .275  (.091-.434) | 9.007  (1,39) | .005 | .188  (.020-.384) | 9.006  (1,39) | .005 | .188  (.020-.384) | 9.714  (1,39) | .003 | .199  (.025-.395) |
|  | Intensity x Condition x Block | 0.251  (1,39) | .619 | .006  (.000-.126) | 0.474  (1,39) | .495 | .012  (.000-.146) | 0.105  (1,39) | .748 | .003  (.000-.076) | 0.827  (1,39) | .369 | .021  (.000-.168) | 0.002  (1,39) | .963 | .000  (.000-.017) | 0.703  (1,39) | .407 | .018  (.000-,160) |
|  | Condition x Block x Repetition | 0.321 (2.602,101.494) | .782 | .008  (.000-.047) | 0.159 (2.991,116.643) | .923 | .004  (.000-.020) | 0.211  (2.929,114.218) | .885 | .005  (.000-.016) | 0.904  (3.273,127.658) | .448 | .023  (.000-.072) | 0.365  (2.588,100.951) | .749 | .009  (.000-.051) | 1.236  (2.723,106.207) | .300 | .031  (.000-.098) |
|  | Intensity x Condition x Block x Repetition | 0.901 (3.681,143.572) | .459 | .023  (.000-.066) | 1.080 (3.622,141.254) | .366 | .027  (.000-.075) | 0.855  (3.743,145.986) | .487 | .021  (.000-.050) | 0.145  (2.806,109.453) | .923 | .004  (.000-.021) | 0.931  (3.821,149.026) | .445 | .023  (.000-.065) | 0.198  (2.781,108.466) | .885 | .005  (.000-.040) |

| c | | Electromyography Measures | | | | | | | | | | | | | | | | | |
| --- | --- | --- | --- | --- | --- | --- | --- | --- | --- | --- | --- | --- | --- | --- | --- | --- | --- | --- | --- |
|  |  | Flexor Carpi Radialis RMS | | | Extensor Carpi Radialis RMS | | | Co-Contraction Index | | | Normalised Flexor Carpi Radialis RMS | | | Normalised Extensor Carpi Radialis RMS | | | Normalised Co-Contraction Index | | |
|  |  | $F$  $(df)$ | $p$ | $\eta_{P}^{2}$  (95%CI) | $F$  $(df)$ | $p$ | $\eta_{P}^{2}$  (95%CI) | $F$  $(df)$ | $p$ | $\eta_{P}^{2}$  (95%CI) | $F$  $(df)$ | $p$ | $\eta_{P}^{2}$  (95%CI) | $F$  $(df)$ | $p$ | $\eta_{P}^{2}$  (95%CI) | $F$  $(df)$ | $p$ | $\eta_{P}^{2}$  (95%CI) |
| Main Effects | Intensity | 47.589  (1,39) | <.001 | .550  (.319,.681) | 6.581  (1,39) | .014 | .144  (.006,.339) | 17.042  (1,39) | <.001 | .304  (.084,.489) | 76.952  (1,39) | <.001 | .664  (.446,.763) | 16.233  (1,39) | <.001 | .294  (.077,.481) | 37.699  (1,39) | <.001 | .492  (.253,.638) |
|  | Repetition | 9.625  (1.722,67.155) | <.001 | .198  (.046,.345) | 6.836  (2.008,78.293) | .002 | .149  (.024,.280) | 4.517  (2.292,89.378) | .010 | .104  (.007,.217) | 10.881  (1.960,76.428) | <.001 | .218  (.066,.355) | 11.068  (1.965,76.637) | <.001 | .221  (.068,.3560 | 5.878  (2.340,91.275) | .002 | .131  (.019,.248) |
| Interaction Effects | Condition x Block | 9.646  (1,39) | .004 | .198  (.024-.394) | 7.642  (1,39) | .009 | .164  (.011-.360) | 16.268  (1,39) | <.001 | .294  (.078-.481) | 10.706  (1,39) | .002 | .215  (.032-.410) | 5.212  (1,39) | .028 | .118  (.000-.310) | 17.217  (1,39) | <.001 | .306  (.086-.491) |
|  | Intensity x Condition x Block | 0.067  (1,39) | .797 | .002  (.000-.094) | 0.155  (1,39) | .696 | .004  (.000-.114) | 3.377  (1,39) | .074 | .080  (.000-.263) | 0.716  (1,39) | .403 | .018  (.000-.161) | 0.445  (1,39) | .509 | .011  (.000-.143) | 2.689  (1,39) | .109 | .065  (.000-.243) |
|  | Condition x Block x Repetition | 0.419  (2.809,109.538) | .726 | .011  (.000-.054) | 0.442  (2.184,85.172) | .661 | .011  (.000-.069) | 0.128  (3.405,132.788) | .958 | .003  (.000-.008) | 0.305  (3.166,123.478) | .832 | .008  (.000-.035) | 0.360  (2.683,104.637) | .760 | .009  (.000-.049) | 0.129  (3.300,128.687) | .954 | .003  (.000-.010) |
|  | Intensity x Condition x Block x Repetition | 1.384  (2.565,100.037) | .254 | .034  (.000-.109) | 1.088  (3.043,118.696) | .357 | .027  (.000-.085) | 2.864  (3.446,134.406) | .032 | .068  (.000-.143) | 1.846  (3.354,130.788) | .135 | .045  (.000-.145) | 0.748  (3.117,121.569) | .530 | .019  (.000-.066) | 2.587  (3.478,135.625) | .047 | .062  (.000-.134) |

| d | | Psychomotor Measures | | | | | | | | | | | | | | |
| --- | --- | --- | --- | --- | --- | --- | --- | --- | --- | --- | --- | --- | --- | --- | --- | --- |
|  | | Sum of Contract False Starts | | | Sum of Relax False Starts | | | Contract Reaction Time | | | Relax Reaction Time | | | Contraction Duration | | |
|  | | $F$  $(df)$ | $p$ | $\eta_{P}^{2}$  (95%CI) | $F$  $(df)$ | $p$ | $\eta_{P}^{2}$  (95%CI) | $F$  $(df)$ | $p$ | $\eta_{P}^{2}$  (95%CI) | $F$  $(df)$ | $p$ | $\eta_{P}^{2}$  (95%CI) | $F$  $(df)$ | $p$ | $\eta_{P}^{2}$  (95%CI) |
| Main Effects | Intensity | 7.972  (1,39) | .007 | .170  (.013-.366) | 2.811  (1,39) | .102 | .067  (.000-.246) | 0.982  (1,34) | .329 | .028  (.000-.195) | 4.952  (1,39) | .032 | .113  (.000-.304) | 4.777  (1,39) | .035 | .109  (.000-.300) |
|  | Repetition | 3.226  (3.327,129.743) | .021 | .076  (.001-.156) | 0.552  (2.471,96.373) | .614 | .014  (.000-.068) | 1.297  (2.845,96.714) | .280 | .037  (.000-0.111) | 11.629  (2.891,112.758) | <.001 | .230  (.094-.340) | 1.835  (3.102,120.992) | .142 | .045  (.000-.115) |
| Interaction Effect | Condition x Block | 0.440  (1.39) | .511 | .011  (.000-.143) | 0.015  (1,39) | .903 | .000  (.000-.062) | 4.944  (1,34) | .033 | .127  (.000-.333) | 7.366  (1,39) | .010 | .159  (.010-.355) | 9.964  (1,39) | .003 | .203  (.027-.399) |
|  | Intensity x Condition x Block | 0.126  (1,39) | .724 | .003  (.000-.109) | 2.711  (1,39) | .108 | .065  (.000-.243) | 0.698  (1,34) | .409 | .020  (.000-.178) | 2.877  (1,39) | .098 | .069  (.000-.248) | 0.008  (1,39) | .929 | .000  (.000-.048) |
|  | Condition x Block x Repetition | 0.733  (3.124,121.836) | .539 | .018  (.000-.065) | 1.400  (3.188,124.331) | .245 | .035  (.000-.100) | 0.800  (3.077,104.622) | .499 | .023  (.000-.076) | 1.645  (3.544,138.233) | .173 | .040  (.000-.100) | 4.878  (3.659,142.718) | .001 | .111  (.019-.195) |
|  | Intensity x Condition x Block x Repetition | 0.367  (4,156) | .832 | .009  (.000-.029) | 0.106  (2.931,114.311) | .954 | .003  (.000-.011) | 0.718  (4,136) | .581 | .021  (.000-.059) | 0.761  (3.687,143.803) | .543 | .019  (.000-.058) | 1.010  (3.311,129.135) | .396 | .025  (.000-.076) |

| e | | Physiological Measures | | | | | |
| --- | --- | --- | --- | --- | --- | --- | --- |
|  |  | Heart Rate | | | Breathing Frequency | | |
|  |  | $F$  $(df)$ | $p$ | $\eta_{P}^{2}$  (95%CI) | $F$  $(df)$ | $p$ | $\eta_{P}^{2}$  (95%CI) |
| Main Effects | Intensity | 10.077  (1,39) | .003 | .219  (.027,.401) | 0.005  (1,39) | .994 | .000  (.000-.038) |
|  | Repetition | 1.570  (2.154,77.545) | .213 | .042  (.000-.138) | 5.324  (3.178,120.754) | .001 | .123  (.022,.219) |
| Interaction Effect | Condition x Block | 1.312  (1,39) | .260 | .035  (.000-.191) | 1.028  (1,39) | .317 | .026  (.000-.178) |
|  | Intensity x Condition x Block | 1.605  (1,39) | .213 | .043  (.000-.204) | 1.158  (1,39) | .289 | .030  (.000-.184) |
|  | Condition x Block x Repetition | 2.149  (1.837,66.119) | .129 | .056  (.000-.174) | 0.408  (2.593,98.518) | .718 | .011  (.000-.056) |
|  | Intensity x Condition x Block x Repetition | 0.929  (1.420,51.123) | .372 | .025  (.000-.144) | 0.615  (3.420,129.977) | .627 | .016  (.000-.055) |

Table S4. Raw peak force regression analysis 1 output with all three models shown.

Table S5. Raw mean force regression analysis 1 output with all three models shown.

Table S6. Raw force-time integral regression analysis 1 output with all three models shown.

# Table S7. Students $t$ test and non-parametric equivalents for comparisons between muscle pain and thermal pain ratings by participant sex.

# Table S8. Raw peak force regression analysis 2 output with all three models shown.

| Model Coefficients - Peak_Force | | | | | | | | | | | | | |
| --- | --- | --- | --- | --- | --- | --- | --- | --- | --- | --- | --- | --- | --- |
|  | | | | | | **95% Confidence Interval** | | | |  | | | |
| **Predictor** | | **Estimation** | | **SE** | | **Lower** | | **Upper** | | **t** | | **p** | |
| Intercept ᵃ |  | 38.449 |  | 4.0908 |  | 30.368 |  | 46.5302 |  | 9.399 |  | < .00001 |  |
| Muscle_Pain |  | -0.757 |  | 0.3477 |  | -1.444 |  | -0.0706 |  | -2.179 |  | 0.03089 |  |
| Intensity: |  |  |  |  |  |  |  |  |  |  |  |  |  |
| 2 – 1 |  | 53.694 |  | 6.0026 |  | 41.836 |  | 65.5523 |  | 8.945 |  | < .00001 |  |
| Muscle_Pain ✻ Intensity: |  |  |  |  |  |  |  |  |  |  |  |  |  |
| Muscle_Pain ✻ (2 – 1) |  | 0.487 |  | 0.3894 |  | -0.283 |  | 1.2558 |  | 1.249 |  | 0.21339 |  |
| MVC_Fatigue |  | -0.379 |  | 0.0783 |  | -0.533 |  | -0.2239 |  | -4.833 |  | < .00001 |  |
| VAS_Fatigue |  | -0.169 |  | 0.1963 |  | -0.557 |  | 0.2185 |  | -0.862 |  | 0.38978 |  |
| ᵃ Represents reference level | | | | | | | | | | | | | |
|  | | | | | | | | | | | | | |

# Table S9. Raw mean force regression analysis 2 output with all three models shown.

| Model Coefficients - Mean_Force | | | | | | | | | | | | | |
| --- | --- | --- | --- | --- | --- | --- | --- | --- | --- | --- | --- | --- | --- |
|  | | | | | | **95% Confidence Interval** | | | |  | | | |
| **Predictor** | | **Estimate** | | **SE** | | **Lower** | | **Upper** | | **t** | | **p** | |
| Intercept ᵃ |  | 24.5476 |  | 2.9481 |  | 18.724 |  | 30.3715 |  | 8.327 |  | < .00001 |  |
| Muscle_Pain |  | -0.5512 |  | 0.2506 |  | -1.046 |  | -0.0562 |  | -2.200 |  | 0.02931 |  |
| Intensity: |  |  |  |  |  |  |  |  |  |  |  |  |  |
| 2 – 1 |  | 38.6757 |  | 4.3259 |  | 30.130 |  | 47.2214 |  | 8.941 |  | < .00001 |  |
| Muscle_Pain ✻ Intensity: |  |  |  |  |  |  |  |  |  |  |  |  |  |
| Muscle_Pain ✻ (2 – 1) |  | 0.3569 |  | 0.2806 |  | -0.197 |  | 0.9113 |  | 1.272 |  | 0.20535 |  |
| MVC_Fatigue |  | -0.2917 |  | 0.0565 |  | -0.403 |  | -0.1802 |  | -5.167 |  | < .00001 |  |
| VAS_Fatigue |  | -0.0734 |  | 0.1415 |  | -0.353 |  | 0.2061 |  | -0.519 |  | 0.60473 |  |
| ᵃ Represents reference level | | | | | | | | | | | | | |
|  | | | | | | | | | | | | | |

# Table S10. Raw force-time integral regression analysis 2 output with all three models shown.

| Model Coefficients - Integral | | | | | | | | | | | | | |
| --- | --- | --- | --- | --- | --- | --- | --- | --- | --- | --- | --- | --- | --- |
|  | | | | | | **95% Confidence Interval** | | | |  | | | |
| **Predictor** | | **Estimate** | | **SE** | | **Lower** | | **Upper** | | **t** | | **p** | |
| Intercept ᵃ |  | 82.554 |  | 10.355 |  | 62.097 |  | 103.0108 |  | 7.972 |  | < .00001 |  |
| Muscle_Pain |  | -1.838 |  | 0.880 |  | -3.576 |  | -0.0989 |  | -2.088 |  | 0.03845 |  |
| Intensity: |  |  |  |  |  |  |  |  |  |  |  |  |  |
| 2 – 1 |  | 125.583 |  | 15.195 |  | 95.566 |  | 155.6006 |  | 8.265 |  | < .00001 |  |
| Muscle_Pain ✻ Intensity: |  |  |  |  |  |  |  |  |  |  |  |  |  |
| Muscle_Pain ✻ (2 – 1) |  | 1.256 |  | 0.986 |  | -0.691 |  | 3.2033 |  | 1.274 |  | 0.20452 |  |
| MVC_Fatigue |  | -0.975 |  | 0.198 |  | -1.367 |  | -0.5836 |  | -4.918 |  | < .00001 |  |
| VAS_Fatigue |  | -0.252 |  | 0.497 |  | -1.233 |  | 0.7300 |  | -0.506 |  | 0.61334 |  |
| ᵃ Represents reference level | | | | | | | | | | | | | |
|  | | | | | | | | | | | | | |

# Material S1. Dijon Physical Activity Score

Rate each of the proposals below using the proposed scale. Circle the number on the right that best matches what is generally true for you.

| 1. Do you consider yourself as:   1. Very active and sporty 2. Moderately physically active 3. Rather not physically active 4. Frankly sedentary | 3  2  1  0 |
| --- | --- |
| 2. Do you consider that your **daily activities** (social and/or professional: travel, handling, DIY, shopping, cleaning, dishes, ironing, etc.) correspond to:   1. Intense physical stress 2. At an average physical stress 3. Moderate physical stress 4. The absence of genuine physical stress | 3  2  1  0 |
| 3. Your **daily activities** (social and/or professional) take up approximately:   1. More than 10 hours per week 2. Between 6 and 10 hours per week 3. Between 2 and 6 hours per week 4. Less than 2 hours per week 5. No time spent per week | 4  3  2  1  0 |
| 4. **The sporting or leisure activity** you carry out is:   1. High intensity (severe muscle fatigue) 2. Moderate intensity (moderate muscle fatigue) 3. Light intensity (without muscle fatigue) 4. You do not exercise | 3  2  1  0 |
| 5. You are used to practicing this **or these physical activities** (sports and leisure):   1. Daily 2. 3 to 6 times a week 3. 1 to 2 times a week 4. Irregularly 5. Never | 4  3  2  1  0 |
| 6. The average duration **of your physical activity** sessions (sports and leisure):   1. Is 60 minutes and more 2. Is 30 to 60 minutes 3. Is 15 to 30 minutes 4. Is less than 15 minutes 5. You do not practice any physical activity | 4  3  2  1  0 |
| 7. How many months per year do you do this **or these physical activities** (sports and leisure)?   1. More than 9 months per year 2. Between 4 and 9 months per year 3. Less than 4 months per year 4. Never | 3  2  1  0 |
| 8. Does **physical activity** (sports and recreation) usually lead to you:   1. Severe fatigue and/or shortness of breath 2. Moderate fatigue and shortness of breath 3. No feelings of fatigue or shortness of breath | 2  1  0 |
| 9. You stay at rest (sleep, naps or awake rest):   1. Less than 12 hours per day 2. Between 12 and 16 hours a day 3. Between 16 and 20 hours a day 4. More than 20 hours a day | 3  2  1  0 |

# Material S2. Edinburgh Manual Laterality Questionnaire

Please indicate which hand you prefer to use for the following activities, indicating a "+" in the appropriate column. Where your preference is so strong that you would never try to use the other hand unless you absolutely have to, write "+ +". In case you are indifferent to the use of either hand to perform the activity, write a "+" in both columns.

Some activities require the use of both hands. In this case, the part of the task or object for which a manual preference is desired is indicated in parentheses.

Please try to answer all questions and leave a blank only if you have no experience with the task or object.

| What hand do you use... | LEFT | RIGHT | What hand do you use… | LEFT | RIGHT |
| --- | --- | --- | --- | --- | --- |
| To write |  |  | To use a knife |  |  |
| To draw |  |  | To use a spoon |  |  |
| To throw a ball |  |  | To comb |  |  |
| To use scissors |  |  | To strike with a hammer |  |  |
| To hold your toothbrush |  |  | To use a screwdriver |  |  |

# Material S3. Task-Specific Motivation Questionnaire

Please answer some questions about your attitude to the task you are about to do (INTERMITTENT ISOMETRIC HANDGRIP TASK). Rate your agreement with the following statements by circling one of the following answers. Make sure you answer every question.

0 = not at all 1 = a little bit 2 = somewhat 3 = very much 4 = extremely

| 1. | I expect the content of the task will be interesting…………………… | 0 | 1 | 2 | 3 | 4 |
| --- | --- | --- | --- | --- | --- | --- |
| 2. | The only reason to do the task is to get an external reward (e.g. payment) | 0 | 1 | 2 | 3 | 4 |
| 3. | I would rather spend the time doing the task on something else……… | 0 | 1 | 2 | 3 | 4 |
| 4. | I am concerned about not doing as well as I can…………………….. . | 0 | 1 | 2 | 3 | 4 |
| 5. | I want to perform better than most people do………………………… | 0 | 1 | 2 | 3 | 4 |
| 6. | I will become fed up with the task……………………………………. | 0 | 1 | 2 | 3 | 4 |
| 7. | I am eager to do well…………………………………………………. | 0 | 1 | 2 | 3 | 4 |
| 8. | I would be disappointed if I failed to do well on the task…………….. | 0 | 1 | 2 | 3 | 4 |
| 9. | I am committed to attaining my performance goals………………….. | 0 | 1 | 2 | 3 | 4 |
| 10. | Doing the task is worthwhile…………………………………………. | 0 | 1 | 2 | 3 | 4 |
| 11. | I expect to find the task boring……………………………………….. | 0 | 1 | 2 | 3 | 4 |
| 12. | I feel apathetic about my performance……………………………….. | 0 | 1 | 2 | 3 | 4 |
| 13. | I want to succeed on the task…………………………………………. | 0 | 1 | 2 | 3 | 4 |
| 14. | The task will bring out my competitive drives……………………….. | 0 | 1 | 2 | 3 | 4 |
| 15. | I am motivated to do the task………………………………………… | 0 | 1 | 2 | 3 | 4 |

# Material S4. Pain Catastrophising Scale

Each of us will have to endure painful experiences. This can be pain associated with headaches, toothache, muscle or joint pain. We often have to endure painful experiences such as illness, injury, dental treatment or surgery.

In this questionnaire, we ask you to describe the kind of thoughts and emotions you have when you have pain. Below are 13 statements describing different thoughts and emotions that may be associated with pain. Please indicate how much you have these thoughts and emotions, according to the scale below, when you have pain.

| **When I have pain...** | Not at all | Somewhat | Moderately | A lot | All the time |
| --- | --- | --- | --- | --- | --- |
| 1. I am afraid that there is no end to pain. | 0 | 1 | 2 | 3 | 4 |
| 2. I feel like I can't continue. | 0 | 1 | 2 | 3 | 4 |
| 3. It's terrible and I don't think it's ever going to get better. | 0 | 1 | 2 | 3 | 4 |
| 4. It's awful and I feel like it's stronger than me. | 0 | 1 | 2 | 3 | 4 |
| 5. I feel like I can't take the pain anymore. | 0 | 1 | 2 | 3 | 4 |
| 6. I'm afraid the pain will get worse. | 0 | 1 | 2 | 3 | 4 |
| 7. I'm just thinking about other painful experiences. | 0 | 1 | 2 | 3 | 4 |
| 8. With concern, I wish the pain would go away. | 0 | 1 | 2 | 3 | 4 |
| 9. I can't help but think about it. | 0 | 1 | 2 | 3 | 4 |
| 10. I'm just thinking how much it hurts. | 0 | 1 | 2 | 3 | 4 |
| 11. I'm just thinking about how much I want the pain to go away. | 0 | 1 | 2 | 3 | 4 |
| 12. There is nothing I can do to reduce the intensity of the pain. | 0 | 1 | 2 | 3 | 4 |
| 13. I wonder if anything bad will happen. | 0 | 1 | 2 | 3 | 4 |

# Material S5. Five Facets of Mindfulness Questionnaire

Please rate each of the following statements by circling the number that best describes your own opinions of what is generally true for you

| 1 | 2 | 3 | 4 | 5 |
| --- | --- | --- | --- | --- |
| Never or Very Rarely True | Rarely True | Sometimes True | Often True | Very Often or Always True |

| Please rate each of the following statements with the number that best describes your own opinion of what is generally true for you | | | | | |
| --- | --- | --- | --- | --- | --- |
| When I’m walking, I deliberately notice the sensations of my body moving. | 1 | 2 | 3 | 4 | 5 |
| I’m good at finding words to describe my feelings. | 1 | 2 | 3 | 4 | 5 |
| I criticize myself for having irrational or inappropriate emotions. | 1 | 2 | 3 | 4 | 5 |
| I perceive my feelings and emotions without having to react to them. | 1 | 2 | 3 | 4 | 5 |
| When I do things, my mind wanders off and I’m easily distracted. | 1 | 2 | 3 | 4 | 5 |
| When I take a shower or bath, I stay alert to the sensations of water on my body | 1 | 2 | 3 | 4 | 5 |
| I can easily put my beliefs, opinions, and expectations into words. | 1 | 2 | 3 | 4 | 5 |
| I don’t pay attention to what I’m doing because I’m daydreaming, worrying, or otherwise distracted. | 1 | 2 | 3 | 4 | 5 |
| I watch my feelings without getting lost in them. | 1 | 2 | 3 | 4 | 5 |
| I tell myself I shouldn’t be feeling the way I’m feeling. | 1 | 2 | 3 | 4 | 5 |
| I notice how foods and drinks affect my thoughts, bodily sensations, and emotions. | 1 | 2 | 3 | 4 | 5 |
| It’s hard for me to find the words to describe what I’m thinking. | 1 | 2 | 3 | 4 | 5 |
| I am easily distracted. | 1 | 2 | 3 | 4 | 5 |
| I believe some of my thoughts are abnormal or bad and I shouldn’t think that way. | 1 | 2 | 3 | 4 | 5 |
| I pay attention to sensations, such as the wind in my hair or sun on my face. | 1 | 2 | 3 | 4 | 5 |
| I have trouble thinking of the right words to express how I feel about things. | 1 | 2 | 3 | 4 | 5 |
| I make judgments about whether my thoughts are good or bad. | 1 | 2 | 3 | 4 | 5 |
| I find it difficult to stay focused on what’s happening in the present. | 1 | 2 | 3 | 4 | 5 |
| When I have distressing thoughts or images, I “step back” and am aware of the thought or image without getting taken over by it. | 1 | 2 | 3 | 4 | 5 |
| I pay attention to sounds, such as clocks ticking, birds chirping, or cars passing. | 1 | 2 | 3 | 4 | 5 |
| In difficult situations, I can pause without immediately reacting. | 1 | 2 | 3 | 4 | 5 |
| When I have a sensation in my body, it’s difficult for me to describe it because I can’t find the right words. | 1 | 2 | 3 | 4 | 5 |
| It seems I am “running on automatic” without much awareness of what I’m doing. | 1 | 2 | 3 | 4 | 5 |
| When I have distressing thoughts or images, I feel calm soon after. | 1 | 2 | 3 | 4 | 5 |
| I tell myself that I shouldn’t be thinking the way I’m thinking. | 1 | 2 | 3 | 4 | 5 |
| I notice the smells and aromas of things. | 1 | 2 | 3 | 4 | 5 |
| Even when I’m feeling terribly upset, I can find a way to put it into words. | 1 | 2 | 3 | 4 | 5 |
| I rush through activities without being really attentive to them. | 1 | 2 | 3 | 4 | 5 |
| When I have distressing thoughts or images, I am able just to notice them without reacting. | 1 | 2 | 3 | 4 | 5 |
| I think some of my emotions are bad or inappropriate and I shouldn’t feel them. | 1 | 2 | 3 | 4 | 5 |
| I notice visual elements in art or nature, such as colours, shapes, textures, or patterns of light and shadow. | 1 | 2 | 3 | 4 | 5 |
| My natural tendency is to put my experiences into words. | 1 | 2 | 3 | 4 | 5 |
| When I have distressing thoughts or images, I just notice them and let them go. | 1 | 2 | 3 | 4 | 5 |
| I do jobs or tasks automatically without being aware of what I’m doing. | 1 | 2 | 3 | 4 | 5 |
| When I have distressing thoughts or images, I judge myself as good or bad depending on what the thought or image is about. | 1 | 2 | 3 | 4 | 5 |
| I pay attention to how my emotions affect my thoughts and behaviour | 1 | 2 | 3 | 4 | 5 |
| I can usually describe how I feel at the moment in considerable detail. | 1 | 2 | 3 | 4 | 5 |
| I find myself doing things without paying attention. | 1 | 2 | 3 | 4 | 5 |
| I disapprove of myself when I have irrational ideas. | 1 | 2 | 3 | 4 | 5 |

# Material S6. Borg & Borg (2002) Category Ratio-100 (CR100) Scale.

# Material S7. Pre-Trial Visual Analogue Scales for Motivation, Boredom, and Fatigue


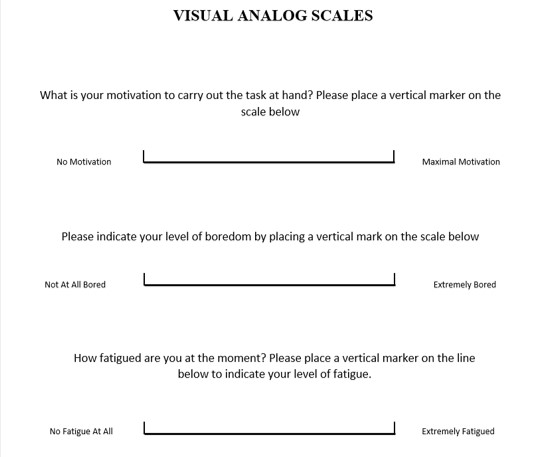


# Material S8. Mid-Trial Visual Analogue Scales for Motivation, Boredom, and Fatigue


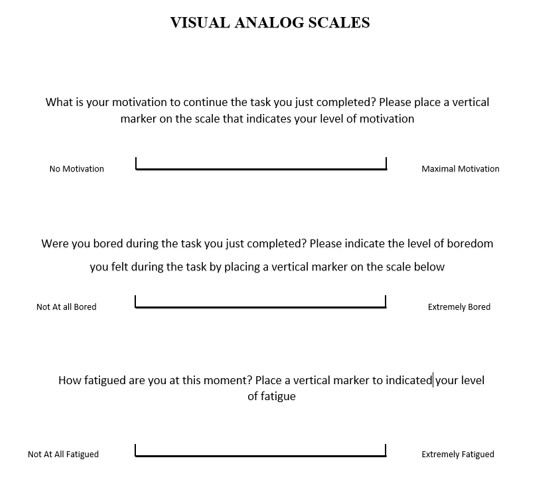


# Material S9. Post-Trial Visual Analogue Scales for Motivation, Boredom, and Fatigue


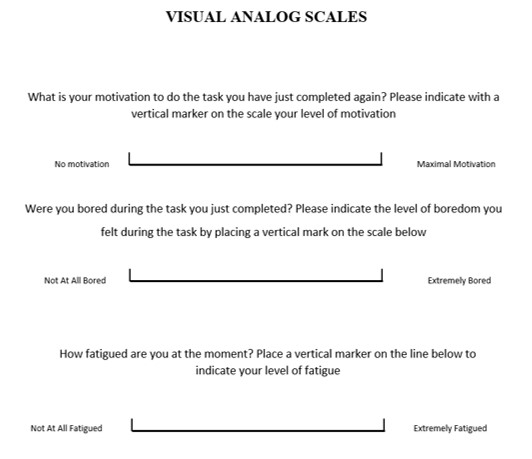


# Material S10. Intensity-Related Effects on Psychological Parameters, Heart Rate, and Breathing Frequency

There was a main effect of intensity on the number of false starts to the contract prompt, reaction time to the relax prompt, and contraction duration with moderate-large effects. There was no intensity main effect for the number of false starts to the relax prompt or reaction time to the contract prompt. There were more false starts to the contract prompt in the light versus strong intensity $(t\_39=2.824,p=.007,d=0.240[0.059-0.420]).$ There was a faster reaction time to the relax prompt in the light versus strong intensity $(t\_39=2.225,p=.032,d=0.142[0.009-0.276]).$ Contraction duration was longer in the light versus strong intensity $(t\_39=2.186,p=.035,d=0.174[0.008-0.341])$.

Intensity main effects were observed for heart rate with a large effect size. Heart rate was higher in the strong versus the light fixed intensity, as expected $(t_{39}=-3.174,p=.003,d=0.241\left[ 0.077-0.405 \right])$. There was no main effect of intensity on breathing frequency.

# Material S11. Influence of Time-on-Task on Pain Stimulation Responses (Condition $\times$ Block $\times$ Repetition Interactions)

A condition $\times$ block $\times$ repetition effect was observed for thermal pain ratings $(F_{2.928,114.208}=10.720, p<.001, \eta_{p}^{2}=.216[.083,.325])$ with a large effect size. There was less difference between experimental and control block 1 thermal pain ratings at repetition 1 $\left( t_{39}=-10.419, p<.001, d=-2.433\left[ -3.882, -0.984 \right] \right)$ versus the same conditions at repetition 5 $\left( t_{39}=-14.465, p<.001, d=-3.483\left[ -5.337, -1.628 \right] \right)$.

There were no significant $\times$ block $\times$ repetition interaction effects for any force or EMG measures. However, exploratory analysis of pairwise comparisons revealed that raw peak force $\left( t_{39}=-1.543, p=.036, d=-0.130\left[ -0.474, 0.214 \right] \right)$, raw mean force $\left( t_{39}=-2.136, p=.036, d=-0.173\left[ -0.507, 0.161 \right] \right)$ and raw force$-$time integral $\left( t_{39}=-2.749, p=.036, d=-0.242\left[ -0.612, 0.128 \right] \right)$ data were all higher during experimental block 1 versus control block 1 at repetition 1 However, there was no difference between the same variables at repetition 5 $\left( p^{'}s>.653,d^{'}s<0.061 \right)$ suggesting that the effect of the thermal pain intervention on most force measures is negated by time-on-task-related fatigue.

A condition $\times$ block $\times$ repetition interaction was observed for contraction duration with a moderate effect size. Pairwise comparisons showed that contraction duration was shorter in control block 1 versus experimental block 1 during repetition 1 $\left( t_{39}=-3.054, p=.016, d=-0.366\left[ -0.875, 0.143 \right] \right)$. Contraction duration did not differ between control block 1 and experimental block 1 at repetition 5 $\left( p=.639,d=0.043 \right)$. There were no condition $\times$ block $\times$ repetition interactions for any false start or reaction time measures. Illustrations of condition $\times$ block $\times$ repetition effects are available in.

# Material S12. Other Exploration of Interaction Effects

Exploratory analysis of probed whether there was a mediating effect of the prescribed fixed perceived effort intensity on experimental pain stimulation across varying time-on-task intensity $\times$ condition $\times$ block $\times$ repetition. The raw co-contraction index measure exhibited a significant intensity $\times$ condition $\times$ block $\times$ repetition main interaction effect $(F_{3.446,134.406}=2.864, p=.032, \eta_{p}^{2}=.068, 95\%CI[.000,.143])$ with a moderate effect size. Likewise, the normalised co-contraction index measure also exhibited a significant intensity $\times$ condition $\times$ block $\times$ repetition main interaction effect $(F_{3.478,135.625}=2.587, p=.047, \eta_{p}^{2}=.062, 95\%CI[.000,.134])$ with a small effect size.

## Material S13. Exercise-Induced Hypoalgesia

Mean thermal pain ratings at rest (calibration) were 51.7 ± 7.3 and 49.1 ± 7.9 for the light and strong intensities respectively. At the same thermal stimulation intensity (i.e., temperature), thermal pain ratings were higher during the pre-exercise calibration than the mean of all thermal pain ratings during experimental block 1 during the light $(t_{1}=6.036,p<.001,d=0.954)$ and strong $(t_{1}=5.835,p<.001,d=0.923)$ intensities. There was no difference between mean thermal pain ratings during the light and strong intensities $(p=.555,d.094)$ inferring that whilst there was exercise-induced hypoalgesia, the perceived intensity of the trials did not have mediatory effect. Repeated measures ANOVAs showed no intensity $\times$ time interactions on mean thermal pain ratings $(p^{'}s>.720)$ at block 1 of light and strong intensities. Therefore, both intensities index the same pattern of thermal pain ratings where ratings at repetition 1 were lower than pre-exercise calibration ratings and despite gradual increases in thermal pain ratings over repetitions, the ratings did not increase back to the initial thermal pain ratings during pre-exercise calibration.

# Material S14. Condition $\times$ Block - Pairwise Corrections

# Material S15. Repetition - Pairwise Corrections.

# Material S16. Intensity $\times$ Condition $\times$ Block - Pairwise Corrections.

# Material S17. Condition $\times$ Block $\times$ Repetition - Pairwise Corrections

All files are available at the project Open Science Framework Page - <https://osf.io/8tbm3/files/osfstorage>
